# Salt hypersensitive mutant 9, a nucleolar APUM23 protein, is essential for salt sensitivity in association with the ABA signaling pathway in *Arabidopsis*

**DOI:** 10.1186/s12870-018-1255-z

**Published:** 2018-03-01

**Authors:** Kai-Chau Huang, Wei-Chih Lin, Wan-Hsing Cheng

**Affiliations:** 1 Institute of Plant and Microbial Biology, Academia Sinica, Taipei, Taiwan; 2 Institute of Plant Biology, National Taiwan University, Taipei, Taiwan

**Keywords:** ABA, APUM23, *Arabidopsis thaliana*, Nucleolus, Proteome, Salt stress, Transcriptome

## Abstract

**Background:**

Although the nucleolus involves two major functions: pre-rRNA processing and ribosome biogenesis/assembly, increasing evidence indicates that it also plays important roles in response to abiotic stress. However, the possible regulatory mechanisms underlying the nucleolar proteins responsive to abiotic stress are largely unknown. High salinity is one of the major abiotic stresses, which hinders plant growth and productivity. Here, genetic screening approach was used to identify a *salt hypersensitive mutant 9* (*sahy9*) mutant, also known as *apum23*, in *Arabidopsis thaliana*. Functional characterization of SAHY9/APUM23 through analyses of gene/protein expression profiles and metabolites was performed to decipher the possible regulatory mechanisms of the nucleolar protein SAHY9/APUM23 in response to salt stress.

**Results:**

Seedlings of the *sahy9/apum23* mutant displayed postgermination developmental arrest and then became bleached after prolonged culture under various salt stresses. Transcriptomic and proteomic analyses of salt-treated *sahy9/apum23* and wild-type seedlings revealed differential expression of genes/proteins that have similar functional categories of biological processes, primarily those involved in cellular and metabolic processes as well as abiotic and biotic stress responses. However, the consistency of differential gene expression at both the transcript and protein levels was low (~ 12%), which suggests the involvement of posttranscriptional processing during the salt response. Furthermore, the altered expression of genes and proteins mediated by SAHY9/APUM23 regarding salt sensitivity involves abscisic acid (ABA) biosynthesis and signaling, abiotic stress responses, and ribosome biogenesis-related genes. Importantly, *NCED3*, *ABI2*, *PP2CA*, and major ABA-responsive marker genes, such as *RD20* and *RD29B*, were down-regulated at both the transcript and protein levels in conjunction with lower contents of ABA and changes in the expression of a subset of LEA proteins in *sahy9/apum23* mutants under salt stress. Moreover, the salt hypersensitivity of the *sahy9/apum23* mutant was largely rescued by the exogenous application of ABA during salt stress.

**Conclusion:**

Our results revealed that SAHY9/APUM23 regulated the expression of ribosome biogenesis-related genes and proteins, which further affected the ribosome composition and abundance, and potential posttranscriptional regulation. The salt hypersensitivity of *sahy9/apum23* is associated with the ABA-mediated signaling pathway and the downstream stress-responsive network of this pathway.

**Electronic supplementary material:**

The online version of this article (10.1186/s12870-018-1255-z) contains supplementary material, which is available to authorized users.

## Background

Salt stress is a major environmental factor, which limits plant growth, development, and productivity. In plants, high salinity generates both ionic toxicity and osmotic stress, which impairs new shoot growth and enhances the senescence of mature leaves, respectively [1]. Plants are sessile organisms that frequently suffer from deleterious environmental stimuli. To cope with abiotic stress, plants can reprogram their gene expression profile, change their signaling pathways, and adjust their metabolic pathways to better suit these adverse conditions. Plants have evolved sophisticated strategies to tolerate high salinity stress, such as increasing osmotic stress tolerance, excluding Na^+^ from leaf blades, and tolerating ion accumulation in tissues [2–4]. Numerous gene functions in salt stress responses and tolerance are induced through complex signal transduction pathways. These pathways include the salt overly sensitive (SOS)-mediated pathway [1, 5, 6], abscisic acid (ABA) biosynthesis and signaling [7], secondary signals (such as reactive oxygen species [ROS] and Ca^2+^) [8–10], the production of osmotic solutes such as proline [11], and small compatible molecules such as Late Embryogenesis Abundant (LEA) proteins [12]. Although great progress has been made in understanding the salt stress response in plants, the detailed regulatory mechanisms largely remain uncharacterized.

Gene expression is regulated at both the transcriptional and posttranscriptional levels, and these levels of regulation are essential for plant growth, development and stress responses [13, 14]. The posttranscriptional regulation of gene expression includes RNA processing, intron splicing, RNA transport and decay, and translation; in general, these processes are collectively referred to as RNA metabolism [14]. RNA-binding proteins (RBPs) are important factors involved in RNA metabolism. Studies of protein structures and functional characterization indicate that diverse organisms exhibit a number of conserved motifs and domains in RBPs, including an RNA-recognition motif (RRM), a zinc finger motif, a K homology (KH) domain, a glycine-rich region, an arginine-rich region, arginine/aspartic acid (RD) repeats, and serine/arginine (SR) repeats [15, 16]. Several nuclear-encoded chloroplast- and mitochondria-targeted RBPs are involved in RNA metabolism in organelles [17–19].

In addition to the aforementioned RBPs, Pumilio proteins are a class of RBP proteins that contain Puf domains, which are conserved across eukaryotes [20]. Puf domains contain multiple tandem repeats, and each repeat is composed of 35–39 amino acids that recognize one RNA base. Pumilio proteins are unique proteins that mostly direct binding to the 3′-untranslated region (UTR) [21]. Pumilio proteins have multifunctional roles, which include involvement in cytoplasmic deadenylation, translational repression, germline stem cell identity maintenance, mitochondria motility and biogenesis, translation initiation, rRNA processing and ribosome biogenesis [20, 22]. There are 25 members of Arabidopsis Pumilio (APUM) proteins identified to date. The expression of these *APUM* genes is tissue-specific and differential. Subcellular localization indicates that these proteins reside in distinct subcellular compartments, including the chloroplast, cytoplasm, and nucleolus, which indicates organelle-specific functions of APUMs [20].

APUM23, a nucleolar protein involved in pre-rRNA processing and regulation of ribosomal gene expression, is constitutively expressed in *Arabidopsis*, particularly in metabolically active and cell-division tissues. Mutation of Arabidopsis *APUM23* results in slow growth, pointed leaves, and defects in venation patterns and leaf structure [23]. The nucleolus is the prominent substructure of the nucleus and functions in rRNA production and ribosome biogenesis/assembly. Moreover, the nucleolus may have multiple functions that are conserved across organisms. For instance, the nucleolus regulates the cell cycle, stress responses, telomerase activity, and aging [24–26]. Compelling evidence suggests that the nucleolus plays a critical role in sensing and responding to stresses. In mammals, diverse stresses can alter the nucleolar structure and protein composition [25]. However, little is known about the involvement of the nucleolus and nucleolar regulatory mechanisms in response to salt stress in higher plants.

To better understand the components involved in the salt-stress response and the affected pathways, we genetically isolated several *salt hypersensitive* (*sahy*) mutants; of which, *sahy9* was identified as a new allele of the *apum23* mutant. Although APUM23 is involved in pre-rRNA processing and ribosome biogenesis [23], the regulatory mechanisms of this protein in response to salt stress remain elusive. In this study, genome-wide analyses of gene expression profiles at both the transcriptomic and proteomic levels revealed that mutation of *SAHY9*/*APUM23* altered the expression of both gene transcripts and proteins that had similar functional classifications based on Gene Ontology (GO) annotation for biological processes. Nevertheless, the consistency of gene expression at both the transcript and protein levels in *sahy9*/*apum23* mutants is low under salt stress. These results suggest that posttranscriptional regulation is involved in the *SAHY9/APUM23*-mediated salt response. The *sahy9*/*apum23* mutants contained lower ABA contents than did the wild type, and the salt hypersensitivity of the mutants under salt stress was largely recovered by exogenous ABA application. Thus, the present study provided molecular, protein, and metabolic data indicating that the nucleolar protein SAHY9/APUM23 regulates salt sensitivity through a mechanism involving the ABA signaling pathway and the downstream stress-responsive or tolerance genes of this pathway.

## Results

### *Salt hypersensitive mutant 9* (*sahy9*) is a new allele of *apum23*

**Fig. 1:**
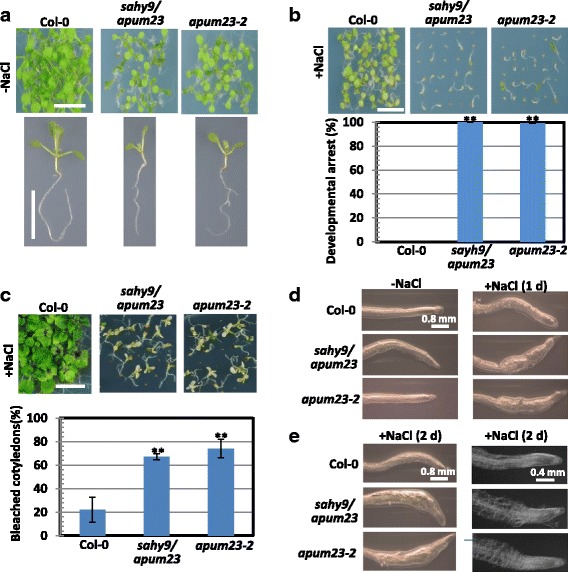
The *sahy9*/*apum23* mutants show salt hypersensitivity. **a**-**b** Phenotypic comparison of plants under normal and salt stress conditions. Seedlings were grown for 10 days on basal medium without (**a**) or with 150 mM NaCl (**b**). The values indicate the means ± SD of three independent experiments, each with 100–150 seeds. **, *P* < 0.01, Student’s *t*-test. Scale = 1 cm. **c** Salt sensitivity. Plants were grown for 30 days on medium supplemented with 150 mM NaCl. The values indicate the means ± SD of three independent experiments, each with 100–150 seeds. **, *P* < 0.01, Student’s *t*-test. Scale = 1 cm. **d**-**e**: Comparison of root tips. The seedlings were grown on basal medium for eight or 9 days, and then transferred to medium supplemented with or without 150 mM NaCl for one (**d**) or two (**e**) days. The images were taken using a confocal microscope (**e**, right panel)

To identify novel components involved in the salt stress response or signaling, a genetic approach was used to screen transfer-DNA (T-DNA) insertion seed pools [27] on agar plates supplemented with 150 mM NaCl; at this concentration, wild-type seeds can grow, but salt-hypersensitive seeds display postgermination developmental arrest and yield bleached seedlings at subsequent growth stages. Ten *salt hypersensitive mutant*s, referred to as *sahy*s, were isolated. Of which, the *sahy9* mutant exhibited striking phenotypes consisting of a small plant size, short roots, and serrated and scrunched leaves (Fig. 1a and Additional file 1: Figure S1b). Amplification of T-DNA-inserted flanking sequences indicated that the *sahy9* mutant had a T-DNA insertion site at the 8th exon of *APUM23* (Additional file 1: Figure S1a), denoted *sahy9/apum23* hereafter. To further confirm whether the *sahy9/apum23* mutant phenotype was due to mutation of the *APUM23* gene, we requested another allelic mutant line, SALK_052992, also known as *apum23–2* [23], from the Arabidopsis Biological Resource Center (ABRC, OH). Both *sahy9/apum23* and *apum23–2* mutant plants had similar phenotypes consisting of a small plant stature and altered leaves when grown on agar plates or in soil (Fig. 1a and Additional file 1: Figure S1b). Furthermore, an RT-PCR assay revealed no detectable *APUM23* transcript in both mutant lines, reflecting that they were knockout mutants (Additional file 1: Figure S1c). These results suggest that the *sahy9* mutant phenotypes are due to defects in the *APUM23* gene.

### Mutation of *SAHY9/APUM23* alters sensitivity to various salts

**Fig. 2:**
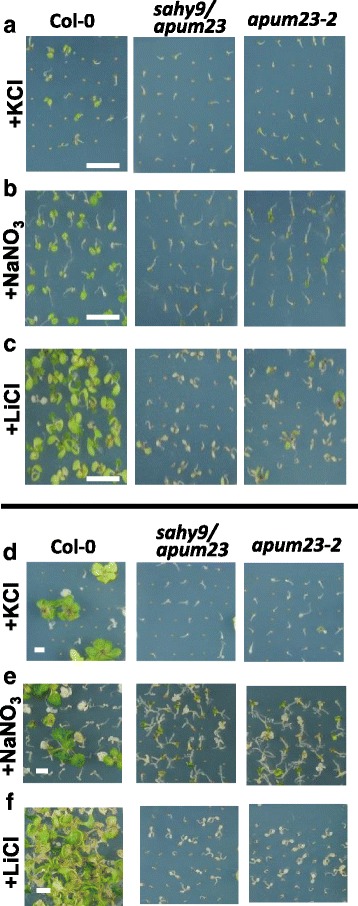
The *sahy9*/*apum23* mutants are sensitive to various salt stresses. **a**-**c** Developmental arrest of the *sahy9* mutant seedlings. Col-0, *sahy9/apum23* and *apum23–2* were grown for 10 days on basal medium supplemented with 150 mM KCl (**a**), 150 mM NaNO_3_ (**b**), or 15 mM LiCl (**c**). **d**-**f** Bleached cotyledons of the *sahy9/apum23* mutants. Seeds were grown on basal medium supplemented with 150 mM KCl (**d**), 150 mM NaNO3 (**e**), and 15 mM LiCl (**f**) for 30, 30, and 20 days, respectively. Three biological repeats, each with approximately 100 seeds, were performed, and consistent results were obtained. Scale = 1 cm

Seedlings of *sahy9/apum23* and *apum23–2* were smaller and had shorter roots than wild-type seedlings when grown for 10 days on agar plates composed of half-strength MS medium supplemented with 1% sucrose (referred to as basal medium) (Fig. 1a); however, these mutants showed postgermination developmental arrest when they grew directly for 10 days on basal medium supplemented with 150 mM NaCl (Fig. 1b). After culture for 30 days on agar plates containing salt, the majority of the mutant seedlings became bleached (died), but the wild-type seedlings largely exhibited steady growth (Fig. 1c). Transferring eight- or nine-day-old mutant seedlings to medium containing salt for one or 2 days resulted in the root tips of mutant plants to appear swollen (Fig. 1d, e), which is associated with irregular cell shapes in the epidermal and cortex cell layers, as observed using a confocal microscope (Fig. 1e, right panel). To further examine whether the salt hypersensitivity observed in *sahy9*/*apum23* and *apum23–2* mutants was specific to NaCl, we grew these mutant seedlings under various salt stress conditions. The results indicated that the *sahy9*/*apum23* and *apum23–2* seedlings were also sensitive to KCl (150 mM), NaNO_3_ (150 mM), and LiCl (15 mM), because the mutants showed postgermination developmental arrest at day 10 (Fig. 2a-c) and more bleached cotyledons at subsequent stages (Fig. 2d-f) compared with wild-type plants. However, developmental arrest and bleached cotyledons were not observed in the *sahy9/apum23* mutants grown on 4% or 6% mannitol-containing media (Additional file 2: Figure S2). Nevertheless, a substantial proportion of the mutant seedlings showed a small plant size and no true leaf development, reflecting that osmotic stress may affect the growth and development of the *sahy9/apum23* mutants. Collectively, these data suggest that the *sahy9*/*apum23* mutants are sensitive to various salt or ion and osmotic stresses.

### Transcriptomic analysis reveals differential expression of genes involved in ABA and stress responses in *sahy9*/*apum23* mutants under salt stress

**Fig. 3:**
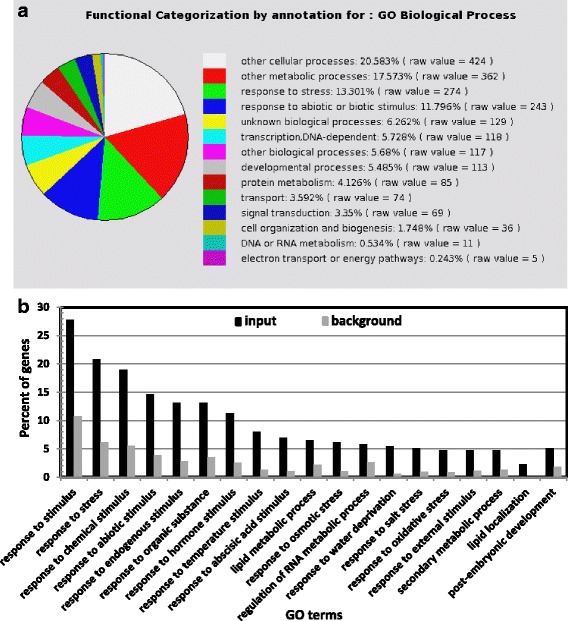
Functional categorization of differentially expressed genes by annotation for GO biological processes. **a** Genes showing altered expression patterns were identified through microarray analysis, and their functions were classified by an analysis of GO biological processes in TAIR. **b** GO enrichment analysis of the differentially expressed genes using agriGO, *P* value < 0.005

**Fig. 4:**
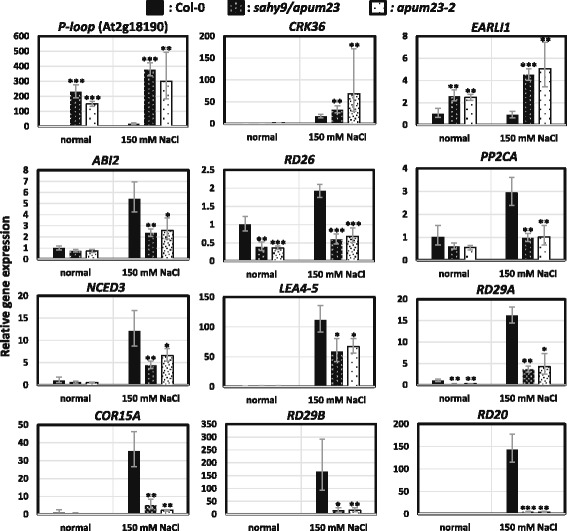
Validation of genes involved in ABA and salt stress responses. The genes used for quantitative real-time PCR (qRT-PCR) were derived from Table 1

To better understand the *SAHY9*-mediated gene expression profiles under salt stress conditions, ten-day-old wild-type and *sahy9*/*apum23* seedlings grown on basal medium agar plates were transferred to agar plates containing 150 mM NaCl for 1 day. The salt-treated seedlings were then harvested and subjected to an Agilent Arabidopsis microarray (Agilent Technology, USA) analysis. After normalization to the wild-type levels, approximately 607 genes (319 up- and 288 down-regulated) were differentially expressed, with a signal fold change ≥3. The analysis of biological processes according to The Arabidopsis Information Resource (TAIR) GO annotations (http://www.arabidopsis.org/tools/index.jsp) indicated that these differentially expressed genes were primarily involved in four functional categorizations: cellular and metabolic processes as well as abiotic and biotic stress responses (Fig. 3a). The genes in these four functional groups constituted approximately 63.3% of the total number of differentially expressed genes, and stress-responsive genes constituted approximately one-fourth (25.1% = 13.3% + 11.8%) of the total number of differentially expressed genes. To gain insight into the GO categories of the differentially expressed genes, a GO enrichment analysis was performed using agriGO (http://systemsbiology.cau.edu.cn/agriGOv2/) [28]. Among the up-regulated genes, the sole GO category was lipid localization [GO:0010876 (2.57e-07)] with input (2.26%) vs. background (0.06%). The major GO category of these differentially expressed genes was response to stimulus, which includes several subcategories, such as response to hormone stimulus and abiotic stress (Fig. 3b). Among these differentially expressed genes, at least 57 were involved in ABA and abiotic stress responses (Table 1), particularly in the salt stress response. For instance, the expression of *NCED3*, a key gene involved in ABA biosynthesis, was decreased 4.4-fold in *sahy9*/*apum23* compared with the wild type. Similarly, the expression of several protein phosphatase genes that participate in the ABA signaling pathway, such as *ABI2*, *PP2CA*, *HAI2*, and *HAI1*, was also down-regulated in *sahy9/apum23*. Several stress response marker genes, such as *RD29A*, *COR15A*, *RD29B*, and *RD20*, were also down-regulated, particularly during salt stress. The expression of 12 genes was verified by quantitative real-time PCR (qRT-PCR) and the results largely showed consistent expression patterns (Fig. 4). Under osmotic stress such as salt stress, plants in deleterious environments can induce the expression of *LEA* proteins. In this study, *LEA* genes were over-represented in the GO category of post-embryonic development (Fig. 3b). In *sahy9*/*apum23* mutants, *LEA* genes, such as *ABR* (AT3G02480), *EM1*/*LEA1* (AT3G51810), *LEA4–5* (AT5G06760), AT2G03850, *COR15A*, and AT2G18340, were down-regulated in *sahy9*/*apum23* compared with the wild type (Fig. 4 and Table 1). This finding further supports the salt hypersensitivity of *sahy9*/*apum23*. Although the proline biosynthesis gene *P5CS1* (AT2G39800) was down-regulated in *sahy9/apum23* under salt stress, the proline contents were not decreased in the mutants compared with the wild type (Additional file 3: Figure S3a). The net product of proline is likely determined by the balance between biosynthesis and catabolism. In addition, no reduction in the P5CS1 protein was observed in our proteomic analysis (see the isobaric tags for relative and absolute quantitation [iTRAQ] data below). Moreover, the expression of two major ABA-independent transcription factors, *DREB2A* and *DREB2B*, showed no significant change in *sahy9/apum23* compared with the wild type (Additional file 3: Figure S3b). These data suggest that the mutation of *SAHY9/APUM23* altered the ABA signaling pathway and the expression of the downstream stress-responsive genes of this pathway.

**Table 1:** Differential expression of genes involved in ABA and abiotic stress responses in *sahy9/apum23*^a^ under salt stress

Locus	Gene name	Fold change^b^	Biological/molecular function
**AT2G18190**	**P-loop containing nucleoside triphosphate hydrolase superfamily protein**	**43.97**	**Response to salt stress**
AT3G28580	P-loop containing nucleoside triphosphate hydrolase superfamily protein	7.85	Response to ABA
**AT4G04490**	**CRK36, CYSTEINE-RICH RLK 36**	**6.89**	**Response to ABA**
AT2G38340	DREB19	5.32	ABA-activated signaling pathway
**AT4G12480**	**EARLI1, EARLY ARABIDOPSIS ALUMINUM-INDUCED 1**	**4.99**	**Response to salt stress**
AT2G15390	FUT4, FUCOSYLTRANSFERASE 4	4.15	Response to salt stress
AT1G67760	TCP-1/cpn60 chaperonin family protein	4.12	Response to salt stress
AT4G28950	ROP9, RHO-RELATED PROTEIN FROM PLANTS 9	4.01	ABA-activated signaling pathway
AT4G11890	Encodes a receptor-like cytosolic kinase, ARCK1	3.96	Response to ABA and salt
AT1G01680	PUB54, PLANT U-Box 54	3.39	Response to stress
AT5G01550	LECRKA4.2	3.35	ABA-activated signaling pathway
AT1G08910	PIAL1, PROTEIN INHIBITOR OF ACTIVATED STAT LIKE 1	3.32	Response to stress
AT4G23260	CRK18, CYSTEINE-RICH RLK 18	3.28	Response to ABA
AT5G01560	LECRKA4.3, LECTIN RECEPTOR KINASE A4.3	3.18	ABA-activated signaling pathway
AT1G43910	P-loop containing nucleoside triphosphate hydrolase superfamily protein	3.10	Response to ABA
AT2G40340	DREB2C	3.01	ABA-activated signaling pathway
AT5G17490	RGL3, RGA-LIKE PROTEIN 3	−3.02	Response to ABA
AT4G01060	CPL3, CAPRICE-LIKE MYB3	−3.05	Response to ABA and salt stress
**AT2G39800**	**P5CS1**	**−3.08**	**Response ABA and salt stress**
AT5G17460	Unknown protein	−3.13	Response to salt stress
AT2G47180	GolS1, GALACTINOL SYNTHASE 1	−3.22	Response to salt stress
AT1G01520	ASG4, ALTERED SEED GERMINATION 4	−3.25	Response to salt stress,
**AT5G57050**	**ABI2**	**−3.37**	**ABA-activated signaling pathway**
AT3G63060	EDL3, EID1-LIKE 3	−3.39	Response to ABA and salt stress
**AT4G27410**	**RD26**	**−3.50**	**Response to ABA**
AT4G25480	DREB1A, CBF3	−3.52	Response to cold and drought
AT5G10230	ANNAT7, ANNEXIN 7	−3.55	Response to salt stress
AT4G05100	MYB74	−3.58	Response to salt stress
**AT3G02480**	**ABR, ABA-RESPONSE PROTEIN; an LEA**	**−3.71**	**Response to ABA**
AT4G21440	MYB102	−3.85	Response to ABA and salt stress
AT2G20880	ERF53, ERF DOMAIN 53	−3.96	Response to salt stress
AT1G43160	RAP2.6, RELATED TO AP2 6	−4.04	Response to ABA and salt stress
**AT3G11410**	**PP2CA**	**− 4.14**	**ABA signaling pathway**
AT1G56600	GOLS2, GALACTINOL SYNTHASE 2	−4.16	Response to salt stress
AT1G54160	NF-YA5, NUCLEAR FACTOR Y, SUBUNIT A5	−4.21	ABA-activated signaling pathway
AT1G69260	AFP1, ABI FIVE BINDING PROTEIN	−4.27	ABA-activated signaling pathway
AT3G28270	AFL1, AT14A-LIKE1	−4.31	Response to water deprivation
**AT3G14440**	**NCED3**	**−4.37**	**ABA biosynthetic process**
AT3G51810	EM1, LEA 1	−4.40	Response to ABA
AT4G25000	AMY1, ALPHA-AMYLASE-LIKE	−4.55	Response to ABA
**AT5G06760**	**LEA4–5**	**−4.57**	**Response to osmotic stress**
**AT5G52310**	**RD29A, RESPONSIVE TO DESICCATION 29A**	**−4.59**	**Response to ABA and salt stress**
AT4G23600	CORI3, CORONATINE INDUCED 1	−4.63	Response to ABA and salt stress
AT1G07430	HAI2, HIGHLY ABA-INDUCED PP2C GENE 2	−4.63	ABA signaling pathway
AT2G03850	LEA family protein	−4.69	
AT5G59220	HAI1	−4.82	ABA signaling pathway
AT3G61890	HB-12, HOMEOBOX 12	−5.12	Response to ABA and salt stress
AT2G47770	TSPO	−5.48	Response to ABA and salt stress
AT5G47450	TIP2;3	−5.50	Response to salt stress
AT5G51760	AHG1, ABA-HYPERSENSITIVE GERMINATION 1	−5.74	Response to ABA
**AT2G42540**	**COR15A, an LEA protein**	**−5.91**	**Response to ABA and salt stress**
**AT5G52300**	**RD29B, RESPONSIVE TO DESICCATION 29B**	**−6.83**	**Response ABA and salt stress**
**AT2G33380**	**RD20**	**−7.22**	**Response ABA and salt stress**
AT2G18340	LEA domain-containing protein	−7.26	
AT2G16005	ROSY1, INTERACTOR OF SYNAPTOTAGMIN1	−8.56	Response to salt stress
AT1G29395	COR413IM1	−9.36	Response to ABA and cold
AT5G24770	VSP2, VEGETATIVE STORAGE PROTEIN 2	−9.81	Response to salt stress

^a^Plants were grown vertically on half-strength MS medium for 10 days and then transferred to fresh medium supplemented with or without 150 mM NaCl for one day. ^b^The fold change in *sahy9/apum23* was normalized against the wild type. The genes in bold font were verified by qRT-PCR and are listed in Fig. 4. The raw data are available at the GEO database under Accession No. GSE99664

### Mutation of *SAHY9*/*APUM23* alters the expression of genes involved in ribosome biogenesis and ribosome abundance under high salinity conditions

**Fig. 5:**
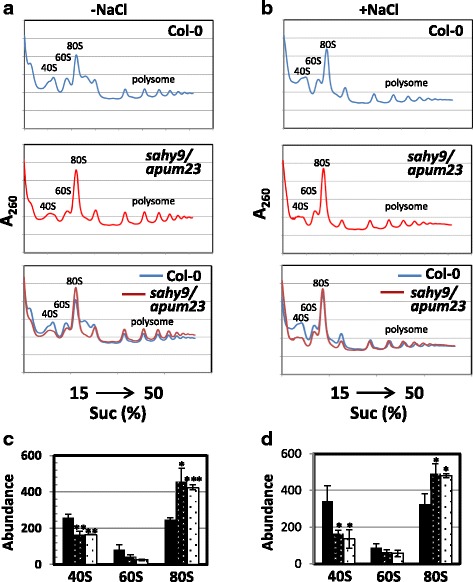
Changes in the ribosome subunit profile in the *sahy9*/*apum23* mutant. **a**-**b** Analysis of the ribosome subunit profile using a sucrose gradient. **c**-**d** Quantified ribosome abundance derived from (**a**) and (**b**), respectively. Plants were grown vertically for 11 days on basal medium with or without 150 mM NaCl for 1 day. The values in (**c**) and (**d**) are the means ± SD of three independent experiments. *, *P* < 0.05; **, *P* < 0.01; ***, *P* < 0.001, Student’s *t*-test

*APUM23* is involved in pre-rRNA processing and regulates the expression of ribosomal protein (RP) and ribosome biogenesis factor (RBF) genes under soil-grown conditions [23]. However, the response of the *APUM23*-mediated expression of RP and RBF genes to salt stress remains unknown. In this study, microarray datasets indicated that at least 20 genes involved in ribosome biogenesis were differentially expressed in *sahy9*/*apum23* compared with the wild type under salt stress (Table 2), and 17 of these differentially expressed genes were up-regulated. *APUM*s belong to a gene family composed of 25 members. The level of *APUM23* transcript in the *sahy9*/*apum23* mutant was 7.7-fold lower than that of the wild type, confirming a defect of the *APUM23* transcript in this mutant. Mutation of *APUM23* resulted in induced expression of other homologous members, such as *APUM7*, *APUM8*, and *APUM12*, suggesting possible genetic redundancy in this gene family. Interestingly, the expression of *RBL19B* was up-regulated in *apum23* mutant plants grown in soil [23], whereas its expression was found to be highly suppressed under salt stress conditions in this study. These results suggest that RP or RBF expression patterns can change under different growth conditions. The expression of six up-regulated genes was verified by qRT-PCR and the results were largely consistent (Additional file 4: Figure S4). In addition, the expression of the verified genes was also up-regulated under normal growth conditions and exhibited a certain degree of difference from the salt treatment, as found for *APUM8* and *NUC-L2*. Furthermore, ribosome profile assays revealed that 40S ribosome abundance decreased, but 80S ribosome levels increased in *sahy9*/*apum23* under both normal and stress conditions (Fig. 5). These results support the important role of *SAHY9*/*APUM23* in ribosome biogenesis/assembly.

**Table 2:** Differential expression of genes involved in ribosome biogenesis in *sahy9/apum23*^a^ under salt stress

Locus	Gene name	Fold change^b^	Biological/molecular function
AT2G03130	Ribosomal protein L12/ ATP-dependent Clp protease adaptor protein	131.75	translation
AT2G18720	Translation elongation factor EF1A/initiation factor IF2 gamma family protein	39.04	translational elongation
AT3G22860	EIF3C-2, EUKARYOTIC INITIATION FACTOR 3C-2	38.10	translational initiation
**AT1G22240**	**APUM8**	**30.13**	**regulation of translation**
AT1G78160	APUM7	22.36	regulation of translation
**AT3G18610**	**NUC-L2, NUCLEOLIN LIKE 2**	**19.76**	**rRNA processing**
**AT1G02830**	**Ribosomal L22e protein family**	**14.24**	**structural constituent of ribosome**
AT1G71770	PAB5, POLY(A)-BINDING PROTEIN 5	12.07	translational initiation
**AT3G28500**	**60S acidic ribosomal protein family**	**11.41**	**structural constituent of ribosome**
AT5G40040	60S acidic ribosomal protein family	8.98	structural constituent of ribosome
AT3G09680	Ribosomal protein S12/S23 family protein	8.92	translation/ structural constituent of ribosome
AT5G56510	APUM12	7.35	regulation of translation
AT4G31520	SDA1 family protein	4.49	ribosomal large subunit biogenesis
**AT2G40010**	**Ribosomal protein L10 family protein**	**4.33**	**structural constituent of ribosome**
**AT5G08600**	**U3 ribonucleoprotein (Utp) family protein**	3.59	rRNA processing
AT5G59240	Ribosomal protein S8e family protein	3.58	ribosome biogenesis, translation
AT5G39850	Ribosomal protein S4	3.03	structural constituent of ribosome
AT1G72340	NagB/RpiA/CoA transferase-like superfamily protein	−3.53	translational initiation (Chloroplast)
AT1G72320	APUM23	−7.7	regulation of translation
**AT3G16780**	RPL19B, RIBSOMAL PROTEIN LIKE 19B	−4001.88	ribosome biogenesis (60S)

^a^Plants were grown vertically on half-strength MS medium for 10 days and then transferred to fresh media supplemented with or without 150 mM NaCl for 1 day. ^b^The fold change in *sahy9/apum23* was normalized against the wild type. The genes in bold font were verified by qRT-PCR and are shown in Additional file 4: Figure S4. The raw data are available at the GEO database under Accession No. GSE99664

### Changes in the protein expression profile of the *sahy9*/*apum23* mutant

**Fig. 6:**
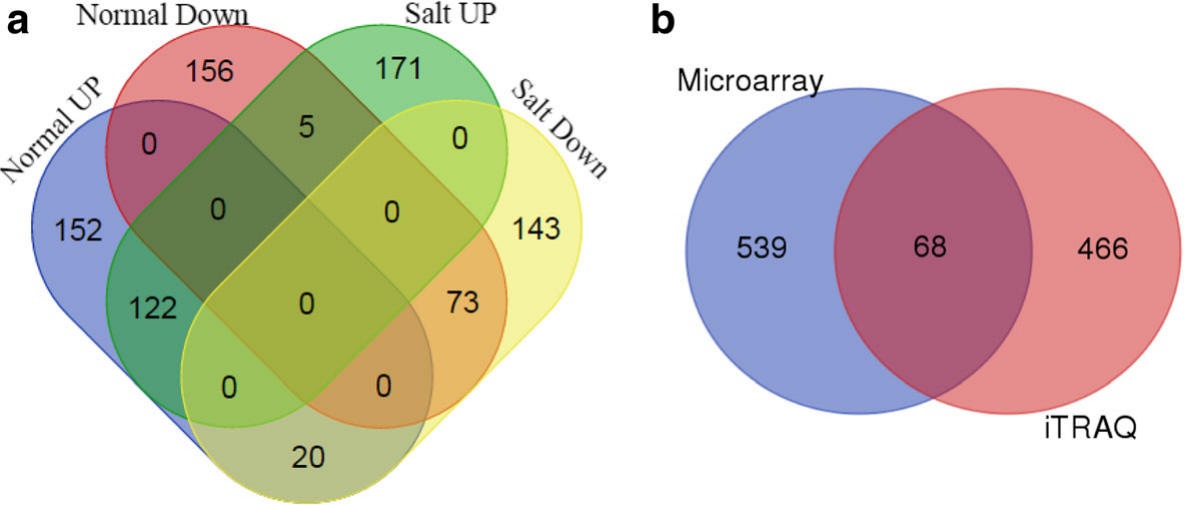
Overlap of identified transcripts and proteins in *sahy9/apum23* under normal or salt stress conditions. **a** Venn diagram representing the overlap of the identified proteins under normal and salt stress conditions. **b** Venn diagram showing the overlap of the expression of transcripts and proteins under salt stress

Because ribosomes participate in protein translation, alteration in ribosome biogenesis and abundance might affect the function of ribosomes in protein translation. Thus, we expected proteomic changes in the *sahy9*/*apum23* mutant. To examine the protein expression profile of the *sahy9*/*apum23* mutant, an iTRAQ analysis was performed to compare the global protein expression profile of 11-day-old *sahy9*/*apum23* mutants and wild-type plants grown on basal medium with or without salt treatment for 1 day. Proteins that were detected in at least two biological replicates of each genotype were selected for expression. Accordingly, 10,550 and 8830 proteins were expressed in *sahy9/apum23* under normal and salt stress conditions, respectively. Proteins with differential expression in *sahy9*/*apum23* was defined by proteins normalized to those of the wild type and having a fold change greater than 1.54 or less than 0.67 (*P* < 0.05, *Z* test). Based on these criteria, the *sahy9*/*apum23* mutant had 528 (294 up-regulated and 234 down-regulated) and 534 (298 up-regulated and 236 down-regulated) differentially expressed proteins under normal and salt stress conditions, respectively (Fig. 6a). Of these proteins, approximately 314 (171 up-regulated and 143 down-regulated) proteins were differentially expressed exclusively under salt stress conditions, and approximately 220 overlapping proteins were expressed under both normal and salt stress conditions. This finding suggests that the expression of approximately 58.8% (314/534) of these proteins was specifically altered in the *sahy9*/*apum23* mutant seedlings under salt stress conditions. A comparison of the transcriptome and proteome indicated that 68 genes showed differential expression at both the transcriptional and protein levels in *sahy9/apum23* mutants under salt stress (Fig. 6b and Additional file 5: Table S1). These genes are largely involved in ribosome biogenesis, ABA and stress responses, carbohydrate metabolism, and lipid metabolism and transport. This low consistency of gene expression (~ 12%) at both the transcript and protein levels suggests an important role of posttranscriptional regulation in *SAHY9/APUM23*-mediated gene expression in response to salt stress.

**Fig. 7:**
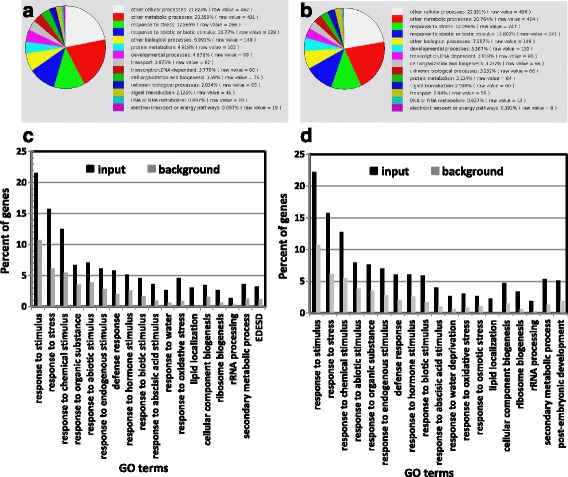
Functional categorization of differentially expressed proteins in *sahy9*/*apum23* compared with the wild type. **a**-**b** Functional classification of the differentially expressed proteins in *sahy9*/*apum23* under normal (**a**) and salt stress (**b**) conditions through analysis of GO biological processes in TAIR. **c**-**d** GO enrichment analysis of differentially expressed genes in *sahy9/apum23*. The seedlings were grown under normal (**c**) or salt stress (**d**) conditions. *P* value < 0.005

The functional classification of these differentially expressed proteins through TAIR GO annotations revealed that these proteins were mostly involved in cellular and metabolic processes and stress response (abiotic and biotic) stimuli, as these proteins constituted 65.5% and 67.0% of all differentially expressed proteins under normal and salt stress conditions, respectively (Fig. 7a, b). To further evaluate the differentially expressed proteins, a GO enrichment analysis was performed. The results indicated that the enriched GO terms belonged to four main GO categories: response to stimulus, cellular component biogenesis, secondary metabolism, and post-embryonic development. These four categories and their prominent corresponding subcategories (or GO terms) are shown in Fig. 7c and d. In general, the enriched GO terms identified for *sahy9/apum23* were similar between normal (Fig. 7c) and salt stress (Fig. 7d) conditions, but they differed somewhat in the input percentage.

### Differential expression of proteins involved in ribosome biogenesis in *sahy9*/*apum23* mutants under normal and salt stress conditions

As mentioned above, one of the four major GO categories was cellular component biogenesis, which included the enriched GO terms “ribosome biogenesis” and “rRNA processing”. Among these differentially expressed proteins, approximately 45 participated in ribosome biogenesis under normal and/or salt stress conditions. Of which, 23 proteins (23/45, 51%) were differentially regulated under both normal and salt stress conditions (Table 3). In contrast, 22 proteins (22/45, 49%) were differentially expressed under either normal or salt stress conditions but not both. This suggests that approximately 50% of differentially expressed proteins involved in ribosome biogenesis can show changes in expression when the *sahy9*/*apum23* mutant seedlings are shifted from normal growth conditions to high saline conditions for 1 day. Interestingly, the comparison of gene expression profiles (Tables 2 and 3) indicated that the expression of only AT5G40040 (60S acidic RP family protein) and AT3G16780 (ribosome protein L19e family protein) was detected at both the transcript and protein levels, reflecting an important role of posttranscriptional regulation in ribosome biogenesis under salt stress conditions.

**Table 3:** Differential expression of proteins involved in ribosome biogenesis in *sahy9/apum23*^a^ under normal and salt stress conditions

Locus	Protein name	Biological/molecular function	Fold change^b^ (*P* value) normal cond.	Fold change (*P* value) salt stress
AT5G40040	60S acidic ribosomal protein family	Structural constituent of ribosome	3.48 (2.59E-09)	3.13 (1.52E-07)
AT4G25630	FIB2, a fibrillarin	rRNA processing	2.17 (0.0002)	3.49 (8.5E-09)
AT5G15550	ATPEIP2, *Arabidopsis thaliana* PESCADILLO ORTHOLOG	rRNA processing	1.94 (0.0017)	2.16 (0.00040)
AT5G18180	H/ACA ribonucleoprotein complex	snoRNA binding	1.93 (0.0019)	2.55 (1.74E-05)
AT4G12600	Ribosomal protein L7Ae/L30e/S12e/Gadd45 family protein	Ribosome biogenesis	1.79 (0.006)	1.83 (0.0056)
AT4G15770	RNA binding protein	Ribosome biogenesis/assembly	1.79 (0.006)	2.09 (0.0007)
AT1G48920	NUC-L1, NUCLEOLIN-LIKE 1	Ribosome biogenesis	1.73 (0.01)	2.01 (0.0013)
AT1G16280	RH36, RNA HELICASE 36	rRNA processing	1.71 (0.011)	1.81 (0.0069)
AT4G36420	Ribosomal protein L12 family protein	Structural constituent of ribosome	1.70 (0.012)	ns
AT3G03920	H/ACA ribonucleoprotein complex	RNA binding, rRNA processing	1.68 (0.014)	2.16 (0.00042)
AT2G24500	FZF, a C2H2 zinc finger protein	Ribosomal large subunit biogenesis	1.68 (0.014)	2.10 (0.00068)
AT5G08180	Ribosomal L7Ae/L30e/S12e/Gadd45 family protein	RNA binding	1.66 (0.017)	1.66 (0.021)
AT5G61330	rRNA processing protein-related		1.62 (0.023)	1.96 (0.0021)
AT3G22660	EBP2, rRNA processing protein-related	Ribosomal large subunit biogenesis	1.61 (0.024)	1.91 (0.0030)
AT3G55620	EIF6A, EMBRYO DEFECTIVE 1624	Ribosomal large subunit biogenesis	1.60 (0.028)	2.11 (0.00063)
AT5G66540	U3 small nucleolar ribonucleoprotein	rRNA processing	1.59 (0.029)	1.74 (0.012)
AT1G63780	IMP4, small nucleolar ribonucleoprotein protein	rRNA processing	1.58 (0.032)	1.62 (0.027)
AT5G20600	rRNA processing-like protein	rRNA processing	1.56 (0.038)	1.77 (0.0088)
AT3G16810	APUM24	RNA binding	1.53 (0.046)	1.73 (0.013)
AT1G13160	ARM repeat superfamily protein	Ribosomal large subunit biogenesis	1.51 (0.051)	2.63 (8.92E-06)
AT1G80750	Ribosomal L30/L7 family protein	Structural constituent of ribosome	1.51 (0.053)	1.93 (0.0026)
AT2G37990	Ribosome biogenesis regulatory (RRS1) family protein	Ribosome biogenesis	ns	1.84 (0.0051)
AT4G25730	FtsJ-like methyltransferase family protein	rRNA processing	ns	2.06 (0.00089)
AT5G14520	PES, PESCADILLO	rRNA processing and ribosome biogenesis	ns	1.84 (0.0051)
AT2G20490	EDA27, EMBRYO SAC DEVELOPMENT ARREST 27	rRNA processing, ribosome biogenesis	ns	1.66 (0.02)
AT5G62190	PRH75, DEAD/DEAH box RNA helicase PRH75	RNA metabolic process	ns	1.74 (0.012)
AT3G19630	Radical SAM superfamily protein	rRNA processing	ns	1.89 (0.0037)
AT2G44860	Ribosomal L24e family protein	Ribosome biogenesis	ns	1.60 (0.033)
AT1G50920	NOG1–1, nucleolar GTP-binding protein	Ribosome biogenesis	ns	1.65 (0.021)
AT1G52930	ATBRX1–2, ARABIDOPSIS HOMOLOGUE OF YEAST BRX1 2	rRNA processing, ribosomal large subunit assembly	ns	1.67 (0.019)
AT3G27180	An SAM-dependent methyltransferase	RNA/rRNA methylation	ns	1.74 (0.011)
AT2G40590	Ribosomal S26e family protein	Structural constituent of ribosome	ns	0.57 (0.0091)
AT3G15460	ATBRX1 1, ARABIDOPSIS HOMOLOGUE OF YEAST BRX1 1	rRNA processing, ribosomal large subunit assembly	ns	1.86 (0.0043)
AT5G10360	RPS6B, RIBOSOMAL PROTEIN SMALL SUBUNIT 6B	Ribosomal small subunit biogenesis	ns	0.64 (0.028)
AT3G43980	Ribosomal S14p/S29e family protein	Structural constituent of ribosome	0.67 (0.046)	ns
AT5G04800	Ribosomal S17 family protein	Structural constituent of ribosome	0.66 (0.041)	ns
AT2G39390	Ribosomal L29 family protein	Structural constituent of ribosome	0.66 (0.040)	ns
AT3G46040	RPS15AD, RIBOSOMAL PROTEIN S15A D	Structural constituent of ribosome	0.66 (0.040)	0.59 (0.012)
AT2G32220	Ribosomal L27e protein family	Structural constituent of ribosome	0.64 (0.0278)	ns
AT4G39200	Ribosomal S25 family protein	Structural constituent of ribosome	0.63 (0.024)	ns
AT3G48960	Ribosomal L13e family protein	Structural constituent of ribosome	0.63 (0.021)	ns
AT5G23740	RPS11-BETA, RIBOSOMAL PROTEIN S11-BETA	Structural constituent of ribosome	0.60 (0.012)	ns
AT1G61580	RPL3B, RIBOSOMAL PROTEIN L3 B	Ribosomal large subunit assembly	0.48 (0.00032)	ns
AT3G28900	Ribosomal L34e superfamily protein	Structural constituent of ribosome	0.45 (9.76E-05)	0.60 (0.016)
AT3G16780	Ribosomal L19e family protein	Ribosome biogenesis	0.310 (1.17E-08)	0.35 (9.07E-07)

^a^Plants were grown vertically on half-strength MS medium for 10 days and then transferred to fresh medium supplemented with or without 150 mM NaCl for one day. ^b^The fold change in *sahy9/apum23* was normalized against the wild type. *Cond.* conditions, *ns* no significance

### Altered expression of proteins involved in ABA and stress responses in the *sahy9*/*apum23* mutants

**Fig. 8:**
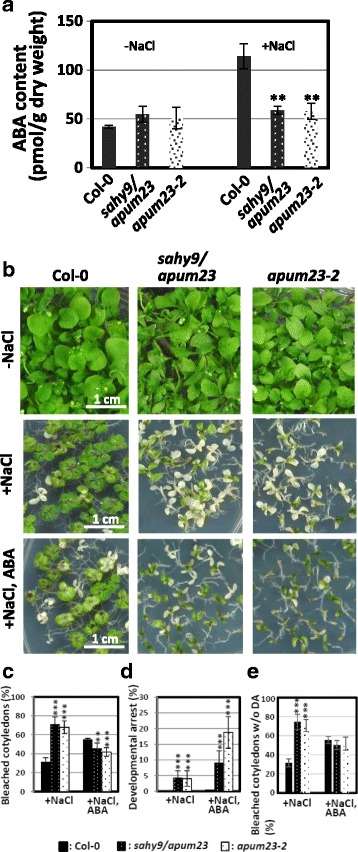
ABA contents and exogenous application of ABA in the *sahy9/apum23* mutant and wild type. **a** ABA contents. Seedlings were grown on basal medium for 10 days, after which they were transferred to basal medium with or without 150 mM NaCl for 1 day. The values indicate the means ± SD of three independent experiments. **, *P* < 0.01, Student’s *t*-test. **b**-**d** Exogenous application of ABA rescues the salt hypersensitivity of *sahy9/apum23* seedlings. Seedlings were grown on basal medium or medium supplemented with 150 mM NaCl and/or 50 nM ABA for 24 days (**b**). Salt hypersensitivity (**c**) and developmental arrest (**d**) were quantified. Salt hypersensitivity (**e**) was derived from the data shown in (**c**) by excluding the developmentally arrested (DA) seedlings from the denominator. The values indicate the means ± SD of three biological repeats, each with 100 seeds. **, *P* < 0.01; ***, *P* < 0.001, Student’s *t*-test

The GO enrichment analysis identified response to stimulus as a major enriched GO term, which included the subcategories: response to ABA and abiotic stress stimuli (Fig. 7c, d). At least 45 proteins involved in the ABA and stress responses were differentially expressed in the *sahy9*/*apum23* mutants compared with wild-type plants grown under normal or salt stress conditions (Table 4). Of which, 20 proteins were significantly up- or down-regulated under both normal and salt stress conditions. These proteins have diverse functions, including in vacuolar storage (cruciferins), in lipid transfer and seed oil body biosynthesis (EARLI1 and OBAP1A), in the defense response (PR4), as a transcription factor (WRKY57), in ion or metal transport (TIP1;1; IRT1), in carbohydrate metabolism (BGLU21, BGLU22, and AMY1), and as a kinase (MPK11). However, 25 of the 45 proteins that show response to ABA and/or osmotic stress, particularly salt stress, were exclusively observed in *sahy9*/*apum23* under salt stress conditions. These 25 proteins included the key ABA biosynthesis protein NCED3, protein phosphatases such as ABI1, ABI2, and PP2CA involved in the ABA signaling pathway, and marker genes of ABA signaling, such as KIN2, RD20, and NHL6. Although KIN1 and RD29B were up-regulated in *sahy9*/*apum23* grown under normal conditions, the expression of these proteins was down-regulated in these plants under high salinity conditions compared with the wild type. This finding supports the salt hypersensitivity of the *sahy9/apum23* mutant seedlings under salt stress conditions. Furthermore, the ABA contents in *sahy9*/*apum23* and *apum23–2* were significantly lower than those in the wild type under salt stress (Fig. 8a); these results are in accordance with the lower expression levels of NCED3 and the downstream ABA-responsive marker proteins. These data support the involvement of the ABA biosynthesis and signaling pathways in the salt hypersensitivity of the *sahy9/apum23* mutants.

**Table 4:** Differential expression of proteins involved in ABA and abiotic stress responses in *sahy9/apum23*^a^ under normal and salt stress conditions

Locus	Protein name	Biological/molecular function	Fold change^b^ (*P*-value) Normal cond.	Fold change (*P*-value) salt stress
AT1G03880	CRU2, CRUCIFERIN 2	Response to ABA	2.76 (1.26E-06)	2.90 (9.79E-07)
AT5G44120	CRU1, CRUCIFERINA	Response to ABA	2.87 (4.76E-07)	2.85 (1.41E-06)
AT3G15353	MT3, METALLOTHIONEIN 3	Response to salt stress	ns	2.78 (2.47E-06)
AT4G28520	CRU3, CRUCIFERIN 3	Response to ABA	2.23 (0.00014)	2.30 (0.00013)
AT3G43700	ATBPM6, BTB-POZ AND MATH DOMAIN 6	Response to salt stress	ns	2.28 (0.00016)
AT1G24120	ARL1, ARG1-LIKE 1	Response to ABA	ns	2.09 (0.00071)
AT4G12480	EARLI 1, EARLY ARABIDOPSIS ALUMINUM-INDUCED 1	Response to ABA and salt stress	2.10 (0.00042)	1.98 (0.0017)
AT5G14920	GASA14	A-STIMULATED IN ARABIDOPSIS 14	1.73 (0.010)	1.96 (0.0021)
AT3G23830	RBGA4, RNA-BINDING GLYCINE-RICH PROTEIN A4	Response to salt stress	ns	1.77 (0.0092)
AT5G03740	HD2C, HISTONE DEACETYLASE 3	Response ABA and salt stress	ns	1.76 (0.0099)
AT2G38310	ATPYL4, PYR1-LIKE 4	ABA-activated signaling pathway	ns	1.72 (0.013)
AT1G05510	OBAP1A, OIL BODY-ASSOCIATED PROTEIN1A	Response to ABA	1.81 (0.0050)	1.66 (0.021)
AT3G04720	PR4, PATHOGENESIS-RELATED 4	Defense and salt response	1.65 (0.019)	1.65 (0.023)
AT5G47450	TIP2;3, TONOPLAST INTRINSIC PROTEIN 2;3	Response to salt stress	ns	1.63 (0.025)
AT4G12470	AZI1, AZELAIC ACID INDUCED 1	Response to cold	ns	1.60 (0.033)
AT1G69310	WRKY57, WRKY DNA-BINDING PROTEIN 57	Response to salt stress	1.52 (0.047)	1.57 (0.040)
AT5G52310	RD29A, RESPONSIVE TO DESICCATION 29A	Response to ABA and salt stress	1.68 (0.014)	ns
AT5G15960	KIN1	Response to ABA and stress	2.04 (0.00069)	0.66 (0.050)
AT2G36830	TIP1;1, TONOPLAST INTRINSIC PROTEIN 1;1	Response to salt stress	0.65 (0.033)	0.66 (0.049)
AT5G26751	ATSK11, SHAGGY-RELATED KINASE 11	Response to salt stress	ns	0.66 (0.046)
AT1G66270	BGLU21, a beta-glucosidase	Response to salt stress	0.67 (0.047)	0.65 (0.041)
AT4G14630	GLP9, GERMIN-LIKE PROTEIN 9	Response to salt stress	0.60 (0.013)	0.64 (0.033)
AT1G69260	AFP1, ABI FIVE BINDING PROTEIN	ABA signaling pathway	ns	0.63 (0.030)
AT1G54100	ALDH7B4, ALDEHYDE DEHYDROGENASE 7B4	Response to ABA and salt stress	ns	0.63 (0.030)
AT5G66400	ATD18, ARABIDOPSIS THALIANA DROUGHT-INDUCED 8	Response to ABA and stress	ns	0.63 (0.030)
AT5G15970	KIN2	Response to ABA and stress	ns	0.62 (0.026)
AT5G02020	SIS, SALT-INDUCED SERINE RICH	Response to salt stress	ns	0.62 (0.022)
AT1G65690	NHL6 (NDR1/HIN1-like 6)	Response to ABA and salt stress	ns	0.62 (0.023)
AT2G37770	AKR4C9, ALDO-KETO REDUCTASE FAMILY 4 MEMBER C9	Response to salt stress	ns	0.60 (0.016)
AT3G50970	LTI30, LOW TEMPERATURE-INDUCED 30	Response to ABA and stress	ns	0.58 (0.010)
AT1G01560	MPK11, MAP KINASE 11	Response to ABA	0.62 (0.020)	0.57 (0.0090)
AT4G26080	ABI1, ABA INSENSITIVE 1	Negative regulator of ABA signaling	ns	0.57 (0.0080)
AT3G22231	PCC1, PATHOGEN AND CIRCADIAN CONTROLLED 1	ABA and defense response	0.34 (1.02E-07)	0.57 (0.0076)
AT5G57050	ABI2, ABA INSENSITIVE 2	Negative regulator of ABA signaling	ns	0.54 (0.0043)
AT2G47770	TSPO, OUTER MEMBRANE TRYPTOPHAN-RICH SENSORY PROTEIN-RELATED	Response to ABA and osmotic stress	ns	0.54 (0.0039)
AT3G22060		Response to ABA	0.65 (0.030)	0.53 (0.0026)
AT5G52300	RD29B, RESPONSIVE TO DESICCATION 29B	Response to ABA and osmotic stress	1.75 (0.0084)	0.51 (0.0018)
AT3G11410	PP2CA, PROTEIN PHOSPHATASE 2CA	Negative regulator of ABA signaling	ns	0.50 (0.0011)
AT1G66280	BGLU22, a beta-glucosidase	Response to salt stress	0.60 (0.011)	0.46 (0.00030)
AT2G33380	RD20, RESPONSIVE TO DESICCATION 20	Response to ABA and stress	ns	0.45 (0.00019)
AT3G26830	PAD3, PHYTOALEXIN DEFICIENT 3	Response to ABA	ns	0.45 (0.00016)
AT1G32350	AOX1D, ALTERNATIVE OXIDASE 1D	Response to ABA	0.64 (0.028)	0.44 (0.00012)
AT3G14440	NCED3, NINE-CIS-EPOXYCAROTENOID DIOXYGENASE 3	Involved in ABA biosynthesis	ns	0.41 (3.28E-05)
AT4G25000	AMY1, ALPHA-AMYLASE-LIKE	Response to ABA	0.44 (7.41E-05)	0.33 (3.25E-07)
AT4G19690	IRT1, IRON-REGULATED TRANSPORTER 1	Response to ABA	0.47 (0.00024)	0.32 (1.17E-07)

^a^Plants were grown vertically on half-strength MS medium for 10 days and then transferred to fresh medium supplemented with or without 150 mM NaCl for one day. ^b^The fold change in *sahy9/apum23* was normalized against the wild type. *Cond.* conditions, *ns* no significance

Another enriched GO term is embryo development ending in seed dormancy (EDESD; Fig. 7c) or post-embryonic development (Fig. 7d), which included a substantial number of proteins belonging to the LEA protein family. Under osmotic stress, plants can produce small molecules such as LEA proteins to protect larger molecules or cellular compartmental membranes from deleterious environmental effects. In this study, 12 LEA proteins, seven of which belong to the LEA 4 group, exhibited different expression patterns between normal and salt stress conditions (Table 5). Although the majority of these LEA proteins were up-regulated in *sahy9*/*apum23* under normal growth conditions, most of them (eight proteins) were down-regulated under salt stress conditions. These results also support the salt hypersensitivity of the *sahy9*/*apum23* mutant seedlings under salt stress conditions.

**Table 5:** Differential expression of LEA proteins in the *sahy9*/*apum23* mutant^a^ compared with the wild type

Locus	Protein name	Biological/molecular function	Fold change^b^ (*P* value) normal conditions	Fold change (*P* value) salt stress
AT1G52690	LEA7 (LEA_4)^c^	Embryo development ending in seed dormancy	2.83 (7.23E-07)	0.36 (2.04E-06)
AT4G21020	(LEA_4)	Embryo development ending in seed dormancy	2.68 (2.61E-06)	2.22 (0.00026)
AT5G44310	(LEA_4)	Embryo development ending in seed dormancy	2.64 (3.73E-06)	2.73 (3.92E-06)
AT3G17520	(LEA_4)	Embryo development ending in seed dormancy	2.60 (5.1E-06)	0.54 (0.0041)
At2G42540	COR15A, (LEA_4)	Response to ABA and cold	2.33 (5.66E-05)	0.60 (0.018)
AT5G53820	LEA		2.31 (7.05E-05)	ns
AT4G02380	ATLEA5 (LEA_3), SAG21	Response to abscisic acid	1.98 (0.0012)	ns
AT5G06760	LEA4–5 (LEA_1)	Response to osmotic stress	1.90 (0.0024)	0.54 (0.0041)
AT3G02480	(LEA_4)	ABR, ABA-RESPONSE PROTEIN	1.86 (0.0033)	0.61 (0.020)
AT3G15670	LEA76 (LEA_4)	Embryo development ending in seed dormancy	1.79 (0.0059)	0.64 (0.035)
AT2G42560	(LEA_4)	Embryo development ending in seed dormancy	1.73 (0.010)	0.33 (1.78E-07)
AT2G42530	COR15B (LEA_4)	Response to ABA and cold	ns	0.55 (0.0056)

^a^Plants were grown vertically on half-strength MS medium for 10 days and then transferred to fresh medium supplemented with or without 150 mM NaCl for 1 day. ^b^The fold change in *sahy9/apum23* was normalized against the wild type. *ns* no significance. ^c^LEA proteins were classified into groups based on previous description [24]

### Exogenous application of ABA largely rescues the salt hypersensitivity of *sahy9/apum23* seedlings under salt stress conditions

As mentioned above, the expression of many proteins involved in both ABA and stress responses were changed in *sahy9/apum23* under salt stress. To further confirm whether the salt hypersensitivity of *sahy9/apum23* is associated with reductions in ABA and its signaling pathway constituents, exogenous ABA was applied to agar plates under salt stress. As shown in Fig. 8b-e, the salt hypersensitive phenotype of *sahy9/apum23* was more intense than that of the wild type under salt stress conditions, even though the seedlings were smaller than the wild-type seedlings; however, exogenous application of ABA (50 nM) largely rescued the salt hypersensitivity of the *sahy9/apum23* seedlings. Instead, the rate of bleached cotyledons in the *sahy9/apum23* seedlings was slightly lower than that in the wild type under 150 mM NaCl + 50 nM ABA conditions (Fig. 8b, c). The lower rate of bleached cotyledons observed in the *sahy9/apum23* mutants was likely due to the slight induction of post-germination developmental arrest (~ 13.4%) in these mutants under NaCl + ABA conditions (Fig. 8d). Thus, examination of only the percentage of expanded and bleached cotyledons in the wild type and mutants (i.e., excluding the developmentally arrested seedlings from the denominator), the rates of bleached cotyledons in the wild type and mutants showed no difference (Fig. 8e). The rate of bleached cotyledons was slightly higher in the wild-type seedlings grown in the presence of NaCl + ABA than in those grown in the presence of NaCl alone. It is likely that the exogenous application of ABA to NaCl-containing medium enhances the intensity of the stress and leads to a slight increase in the rate of bleached cotyledons in the wild-type plants. Furthermore, transcriptional analyses indicated that the expression of *NCED3* was slightly higher in *sahy9/apum23* than in the wild type under NaCl + ABA conditions (Additional file 6: Figure S5). Although transcripts of three *PP2C*s (*ABI1*, *ABI2*, and *PP2CA*) and four stress-responsive genes (*RD29A*, *COR15A*, *RD26*, and *RD20*) were slightly increased in the mutants under NaCl + ABA conditions compared with NaCl conditions, their expression levels were still lower than those in the wild type. Interestingly, the expression of *RD29B* and three *LEA* genes (*LEA4–5*, *LEA7*, and At3g17520) were higher in the mutants than in the wild type under salt stress, and the induced expression of these genes became more pronounced in the mutants under NaCl + ABA conditions. Furthermore, although the ABA contents in *sahy9/apum23* were lower than in the wild type under NaCl conditions, the ABA contents showed no difference between wild type and the mutants under NaCl + ABA conditions (Additional file 7: Figure S6). These data indicate that *SAHY9/APUM23*-mediated salt sensitivity is associated with the ABA signaling pathway together with its downstream responsive genes and that the small plant size of *sahy9/apum23* mutants is likely due to the effects of other pathways.

## Discussion

### Changes in the expression of ribosome biogenesis-related genes and ribosome abundance in *sahy9*/*apum23* under normal and salt stress conditions

SAHY9/APUM23 is a nucleolus-localized protein that functions in pre-rRNA processing and ribosome biogenesis [23]. In this study, GO enrichment analyses of the transcriptome and proteome datasets revealed that the differentially expressed genes identified in *sahy9/apum23* were over-represented in one of the major GO categories: cellular component biogenesis. Within this category, the main sub-categories involved were the regulation of RNA metabolic processes (Fig. 3) and ribosome biogenesis and rRNA processing (Fig. 7). A transcriptomic analysis has indicated that at least 43 genes relative to ribosome biogenesis are differentially expressed in the *apum23* mutants grown in the soil for 3 weeks [23]. However, in the present study, at least 20 genes involved in ribosome biogenesis were differentially expressed in the *sahy9*/*apum23* seedlings grown on agar plates supplemented with NaCl. Of these 20 genes, eight (40%, 8/20) overlapped with those previously identified (Table 2 in this study vs. Table 1 of [23]). Moreover, proteomic analyses revealed that at least 45 proteins involved in ribosome biogenesis were differentially expressed in *sahy9*/*apum23* seedlings grown on normal or NaCl-treated agar plates. Of which, approximately 50% of ribosome biogenesis-related genes can be activated or suppressed under normal or salt stress conditions. Changes in these ribosome biogenesis-related genes and/or proteins under distinct environments could alter ribosome biogenesis/assembly and lead to differences in ribosome subunit abundance (Fig. 5). Altered ribosome profiles have also been reported regarding the mutation of *DIG6* (drought-inhibited growth of lateral roots), which encodes a large 60S subunit nuclear export GTPase1 involved in ribosome biogenesis [29]. Changes in ribosome biogenesis/assembly and abundance may further affect protein translation. The majority of the RP or RBF mutants exhibit auxin-mediated developmental defects in leaf morphology, venation patterning, and root growth (Fig. 1 and Additional file 1: Figure S1). These mutants are presumably due to changes in ribosome composition and further cause preferential translation or undertranslation of certain auxin-related genes, such as *PIN*s [23, 30–32]. However, such changes in genes involved in auxin transport and perception are largely not detectable through transcriptomic and proteomic analyses (in this study; [29]). It is likely that the expression of these auxin-related genes is too low to be detected under these experimental conditions and that only abundant transcripts or proteins can be detected.

In addition to its two major roles in pre-rRNA and ribosome biogenesis/assembly, the nucleolus has been proposed to function in multiple processes, such as the cell cycle and stress responses [25, 26]. Our data provided evidence that the expression of ABA- or abiotic stress-related genes was up-regulated by salt stress (Fig. 4). However, the induction ratios of these genes were lower in *sahy9/apum23* than in the wild type, which suggests that mutation of the nucleolar protein SAHY9/APUM23 affects transcriptional and posttranscriptional regulation both sensitively and widely. The multiple functions of the nucleolus are also reflected by its high heterogeneity, including modification of rRNA, variation in the RP composition and dynamic compositional changes in response to cellular cues or environmental stimuli [26, 33, 34]. In the Arabidopsis genome, each RP and its homologs form a multimember family. Additionally, different tissues, developmental stages, and environmental stimuli may activate the expression of distinct subsets of RPs (in this study and [35]). Thus, the data obtained from the present study may complement the previous report [23].

### Genome-wide analyses of gene expression reveal similar functional categories but low consistency of transcript and protein profiles in *sahy9*/*apum23* mutants under salt stress conditions

Transcriptomic analyses indicated that approximately 607 genes were differentially expressed under salt stress and that changes in the expression of approximately 534 proteins were detectable via iTRAQ analysis under the same growth conditions. Although these differentially expressed genes/proteins had similar functional classifications primarily consisting of cellular and metabolic processes as well as biotic and abiotic stress responses, the changes in transcript and protein profiles showed little overlap. Only 68 genes were differentially expressed at both the transcript and protein levels in *sahy9/apum23* mutants under salt stress conditions. For instance, 20 genes and 36 proteins involved in ribosome biogenesis/assembly showed differential expression in *sahy9/apum23* under salt stress, but only two genes, the 60S acidic RP (AT5G40040) and RPL19e (AT3G16780), were expressed at both the transcript and protein levels (Table 2 vs. Table 3). In addition, 57 transcripts (Table 1) and 45 proteins (Table 4) were differentially expressed and involved in ABA and abiotic stress responses in *sahy9*/*apum23* under salt stress. Among these, only six genes, *ABI2*, *PP2CA*, *RD20*, *NCED3*, *RD29B*, and *AMY1*, were detected at both the transcript and protein levels. This low consistency in the changes in RNA and protein profiles might be due to the following reasons. First, low-abundance proteins, such as membrane-associated proteins, might not be detected under the applied experimental conditions, whereas the transcript levels of the corresponding proteins could show significant changes. Second, high-abundance proteins might exhibit low rates of protein degradation or important housekeeping functions, but their transcript levels may not have met the criterion of a three-fold change expression applied to the transcriptomic analysis. Finally, the differential expression of gene transcripts may not have been efficiently translated to the proteins due to the regulation of posttranscriptional processing. In addition to salt stress, as observed in the present study, a low congruency of transcript and protein profiles has also been detected during ion starvation [36] and light morphogenesis [37]. Thus, posttranscriptional processing or RNA metabolism plays important roles in gene regulation that predominantly affects plant growth and stress responses.

### SAHY9/APUM23 regulates salt sensitivity in association with the ABA signaling pathway and ABA-mediated downstream stress-responsive or tolerance genes

Although the *APUM23* gene function has been well characterized [23], its response to salt stress, particularly as a nucleolar protein, remains unknown. Arabidopsis nucleolin 1 (NUC1) is also an RBP predominantly localized in the nucleolus and functions in pre-rRNA processing, ribosome biogenesis, and plant normal growth [38, 39]. Mutation of *NUC1* (i.e., *parl1*) results in plants with slowed growth and auxin-mediated developmental defects [31]. Rice *NUC1* (*OsNUC1*) is transcriptionally regulated by salt stress, and the overexpression of *OsNUC1* in *Arabidopsis* or rice leads to salt stress tolerance [40]. Because the expression of several ABA biosynthesis and signaling genes, including *NCED3*, *ABI1*, *ABF3*, *RD29A*, and *KIN1*, is down-regulated in *OsNUC1* overexpressors under salt stress conditions [41], the relationship between ABA-mediated gene expression and salt-resistant phenotypes remains to be illustrated. Salt stress increases the calcium ion (Ca^2+^) concentration in the cytosol of plant cells, which further results in activation of calcineurin B-like proteins (CBLs) and CBL-interacting protein kinases (CIPKs). Subsequently, CBL-CIPK mediates the SOS pathway to increase the tolerance of plant cells to salt stress [42]. Although many genes that were identified as differentially expressed in *sahy9*/*apum23* under salt stress in this study were classified as being involved in response to ABA or abiotic stress, the expression of the SOS components (SOS1, SOS2, and SOS3) in *sahy9*/*apum23* did not differ from that of the wild type. In addition, although salt stress induces the production of the osmotic solute proline in plants, the proline contents in *sahy9/apum23* also showed levels similar to those found in the wild type. Thus, the SOS pathway and proline content are not likely involved in SAHY9/APUM23-mediated salt sensitivity.

ABA is the major regulator of abiotic stress resistance and coordinates a complex regulatory network to adapt to osmotic stress [43, 44]. The core ABA signaling pathway is composed of three protein classes: PYR/PYL/RCAR receptors, protein phosphatase 2Cs (PP2Cs), and SNF1-related protein kinase 2 s (SnRK2s). PP2Cs are transcriptionally regulated by ABA [45]. In this study, the PP2C proteins ABI1, ABI2, and PP2CA were down-regulated in *sahy9/apum23* under salt stress (Table 4). Because PP2Cs function as negative regulators [46, 47], a reduction in these PP2C proteins might presumably activate SnRK2s, further triggering downstream ABA-responsive gene expression. Instead, the expression of several ABA-responsive marker genes, such as *KIN1*, *RD29A*, *RD29B* and *RD20*, were reduced at the transcript and/or protein level in the mutant under salt stress conditions. A previous study proposed the existence of two ABA signaling pathways: the *ABI1*-dependent and *ABI1*-independent pathways [48]. Thus, our data support the notion that the SAHY9/APUM23-mediated salt response most likely occurs through an *ABI1*-independent pathway. Furthermore, several lines of evidence also support SAHY9/APUM23-mediated salt sensitivity through the ABA signaling pathway, including reduced expression levels of *NCED3* and a subset of *LEA*s, and lower ABA contents in *sahy9*/*apum23* compared with the wild type under salt stress. Moreover, exogenous ABA application largely rescued the salt-hypersensitive phenotype together with induction of *NCED3* expression and ABA contents in the *sahy9/apum23* mutants under NaCl + ABA conditions.

Because LEA structures are highly hydrophilic and natively unfolded, they may interact with other large molecules to stabilize them against deleterious stress conditions [12, 49]. Most LEAs, including dehydrin RAB18, can be induced by ABA and osmotic stress [50–52]. Furthermore, overexpression of *LEA*s in transgenic plants of rice, *Arabidopsis*, or mustard (*Brassica juncea*) confers tolerance to drought and/or salt stress [53–55]. Consistently, in this study, exogenous ABA application partially rescued the salt-hypersensitive phenotype of the *sahy9/apum23* mutants and led to notably induced expression of three *LEA*s (*LEA4–5*, *LEA7*, and At3g17520) in the mutants relative to the wild type under NaCl + ABA conditions. Notably, the expression of *LEA4–5* in the mutants was lower than that in the wild type after short-term (one-day) salt treatment. Nevertheless, long-term (24-day) salt treatment increased *LEA4–5* expression to a level higher than that in the wild type. A similar expression pattern was also observed for *LEA7* and At3g17520 (Table 5 vs. Additional file 6: Figure S5). These data suggest that LEA proteins might play an important role in adaptation to salt stress. Moreover, with the exception of *RD29B*, which was highly induced, several ABA-mediated stress responsive genes, such as *RD29A*, *RD26*, *RD20* and *COR15A*, were only induced slightly under NaCl + ABA conditions. This differentially induced expression of the canonical stress marker genes also supports the involvement of the ABA-mediated ABI-independent signal pathway in the *sahy9/apum23* mutants in response to salt stress.

## Conclusions

In conclusion, analyses of gene expression profiles and metabolites were performed in the present study to characterize the possible regulatory mechanisms of the nucleolar protein SAHY9/APUM23 in response to salt stress. Gene/protein expression profiles revealed changes in the differential expression of genes or proteins primarily involved in ribosome biogenesis and ABA biosynthesis and signaling as well as changes in the differential expression of both stress-responsive marker genes and a subset of LEA proteins in *sahy9/apum23* in response to salt stress. The altered expression of ribosome biogenesis-related genes or proteins in *sahy9/apum23* might alter the composition and abundance of ribosomes, further affecting the translation of proteins with distinct functions and causing pleiotropic phenotypes. Among these phenotypes, the salt hypersensitivity of *sahy9/apum23* is associated with the ABA-mediated signaling pathway and its downstream stress-responsive network.

## Methods

### Plant materials and growth conditions

*A. thaliana* ecotype Columbia (Col-0) plants were used in this study. Seeds were sterilized and subjected to cold pretreatment at 4 °C for 3 days in the dark, after which they were sowed on agar plates or in soil on the first day of germination. The basal medium used in this study was composed of half-strength MS basal salt [56], B5 organic compounds [57], 0.05% MES [2-(N-morpholino)ethanesulfonic acid monohydrate], and 1% sucrose. The medium was adjusted to pH 5.7, followed by the addition of 7 g/L phytoagar (Duchefa Biochemie, Haarlem, the Netherlands) prior to autoclaving. Unless stated otherwise, the seeds were germinated at 22 °C under a 16/8 h day/night photoperiod and a light intensity of approximately 80 μmol m^− 2^ s^− 1^. The plant materials used in this study was approved by the Academia Sinica Biosafety Review & Biomaterials and Lab Biosafety Information System.

### Genetic isolation of *sahy9*

For the genetic screening of salt-responsive mutants, T-DNA insertion seed pools [27] consisting of more than 10,000 lines were requested from the ABRC. Approximately 400,000 seeds were grown on basal agar medium supplemented with 150 mM NaCl, a concentration in which wild-type plants can germinate and steadily grow. Seedlings that displayed postgermination developmental arrest at day 10 and subsequent bleaching of the cotyledons after three- or four-week culture were referred to as *salt hypersensitive* (*sahy*) mutants. Approximately 10 *sahy* mutants were isolated through this genetic approach. Of which, *sahy9* exhibited a unique phenotype consisting of slow growth, small plant size, and pointed leaves. The *sahy9* mutant was further characterized for its function.

### Microarray analysis

Col-0 and *sahy9*/*apum23* seedlings were grown on basal medium for 10 days, after which they were transferred to medium supplemented with 150 mM NaCl for 1 day. After salt treatment, the seedlings were harvested for total RNA extraction using the RNeasy Plant Mini Kit (Qiagen, Germany). After RNA labeling and hybridization, the GeneChip (Agilent, Arabidopsis 4 × 4.4 K V4) was scanned in accordance with the Agilent standard protocol. The resulting CEL files were analyzed using GeneSpring GX V11.5 software (Agilent). The data were normalized using MAS V5.0 and filtered based on expression levels, employing a raw signal value of 100 as the cutoff value, at least in the wild type or the *sahy9/apum23* mutant. The filtered genes were statistically analyzed using the unpaired *t*-test (*P*-value cutoff of 0.05) and multiple testing corrections in accordance with the Benjamini-Hochberg false discovery rate (FDR) [58]. A three-fold signal change in gene expression was defined as differential expression. Two biological experiments were performed in this study. The raw data are available in the Gene Expression Omnibus (GEO) database under Accession No. GSE99664.

### iTRAQ analysis

Seedlings harvested after 11 days of growth on agar plates with or without 150 mM NaCl treatment for 1 day were used for total protein extraction, which was followed by iTRAQ analysis. Protein sample preparation and iTRAQ analysis were performed as previously described [36]. The final proteomic data were derived from three biological experiments, each with three technical replicates.

### Quantitative RT-PCR

Total RNA was extracted from 11-day-old seedlings grown on agar plates with or without 150 mM NaCl treatment for 1 day, using the RNeasy Plant Mini Kit (Qiagen). Subsequently, 2 μg of DNase I (Qiagen)-treated total RNA was reverse-transcribed with 2 μg of an oligo dT primer using Superscript III Reverse Transcriptase (Invitrogen). qRT-PCR was performed using Power SYBR Green PCR Master Mix (Applied Biosystems) and an Applied Biosystems 7500 Real-Time PCR System. Primer Express v2.0 software (Applied Biosystems) was used to design the primers (Additional file 8: Table S2). The relative transcript levels of the genes were determined via the comparative threshold cycle (C_T_) method using *PP2A* (At1g13320) as the internal control. All experiments were performed in three biological replicates, each with three technical repeats.

### Ribosome profile analyses

Ribosome profile analyses were performed as previously described [37]. In general, 0.3-g samples of 11-day-old seedlings grown on agar plates with or without 150 mM NaCl treatment for 1 day were used for ribosome or polysome extraction using a buffer composed of 200 mM Tris(hydroxymethyl)aminomethane hydrochloride (Tris-HCl) (pH 8.5), 50 mM KCl, 25 mM MgCl_2_, 100 μg/mL heparin, 50 μg/mL cycloheximide, 400 U/mL RNasin (Promega, Madison, WI, USA), 2% polyoxyethylene 10-tridecyl ether, and 1% deoxycholic acid. The mixture was incubated on ice for 5 min, followed by centrifugation at 15000 *g* for 5 min at 4 °C. Thereafter, the supernatant was collected and the pellet discarded. A total of 300 μL of the supernatant was loaded onto a 10-mL continuous sucrose gradient (15–50%) prepared with a gradient maker (ISCO, Lincoln, NE), after which the mixture was centrifuged at 35000 *g* for 3.5 h at 4 °C. The distribution of nucleic acids was detected based on the 254-nm UV absorbance profile (Brandel BR-188, Gaithersburg, MD, USA).

### ABA and proline assays

For the ABA assays, eleven-day-old seedlings grown on agar plates with or without 150 mM NaCl treatment for 1 day were harvested and subsequently subjected to ABA extraction as described previously [59]. ABA was quantified using enzyme-linked immunosorbent assay (ELISA) (Phytodetek ABA kit; Agdia) in accordance with the manufacturer’s recommended protocol. The protocol for the proline assays followed the description provided in previous reports [60].

## Additional files

Additional file 1: **Figure S1.** *SAHY9*/*APUM23* gene structure showing the T-DNA insertion sites. a: Exon-intron structure of SAHY9/APUM23 and T-DNA insertion sites in the mutant lines. b: Phenotypic comparison between the wild-type and mutant lines. Plants were grown in soil for 35 days. c: RT-PCR analysis of the *APUM23* transcript in the wild-type and mutant plants. (PPTX 531 kb)

Additional file 2: **Figure S2.** Effect of osmotic stress on *sahy9/apum23* mutant plants. a-b: Seedlings were grown on basal medium supplemented with 4% (a) or 6% (b) mannitol for 10 or 30 days, respectively. (PPTX 482 kb)

Additional file 3: **Figure S3.** qRT-PCR results of stress-responsive genes and proline contents. a: qRT-PCR of the *P5CS1* gene and proline contents. b: qRT-PCR of stress-responsive genes. Plants were grown on basal medium for 10 days, followed by treatment with 150 mM NaCl for 1 day. *, *P* < 0.05; **, *P* < 0.01, Student’s *t*-test. (PPTX 2453 kb)

Additional file 4: **Figure S4.** Validation of the expression of genes involved in ribosome biogenesis in *sahy9*/*apum23* under salt stress conditions. The verified genes are listed in Table 2. (PPTX 12701 kb)

Additional file 5: **Table S1.** Overlap of differential gene expression at both the transcript and protein levels in the *sahy9/apum23* mutants under salt stress conditions. (DOCX 26 kb)

Additional file 6: **Figure S5.** Expression of genes regulated by ABA and salt stress. Plants were grown on basal medium supplemented with 150 mM NaCl or 150 mM NaCl + 50 nM ABA for 24 days. qRT-PCR was performed for the detection of relative gene expression levels. (PPTX 29522 kb)

Additional file 7: **Figure S6.** ABA contents. Seedlings were grown on basal medium supplemented with NaCl or NaCl + ABA for 24 days. The values indicate the means ± SD of four independent experiments. ***, *P* < 0.001, Student’s *t*-test. (PPTX 88 kb)

Additional file 8: **Table S2.** Primer sequences used in this study. (DOCX 16 kb)

## Abbreviations

ABA: Abscisic acid
ABI: Abscisic acid insensitive
ABR: ABA-RESPONSE PROTEIN
APUM: Arabidopsis Pumilio protein
CBL: Calcineurin B-like protein
CIPK: CBL-interacting protein kinase
ELISA: Enzyme-linked immunosorbent assay
GO: Gene Ontology
iTRAQ: isobaric tags for relative and absolute quantitation
LEA: Late Embryogenesis Abundant Protein
NCED3: Nine-cis-epoxycarotenoid dioxygenase 3
NUC: Nucleolin
P5CS1: DELTA1-PYRROLINE-5-CARBOXYLATE SYNTHASE 1
PP2CA: Protein phosphatase 2CA
PYR/PYL/RCAR: Pyrabactin resistance/PYR1-like/regulatory components of the ABA receptor
qRT-PCR: quantitative real-time polymerase chain reaction
RBF: Ribosome biogenesis factor
RBP: RNA-binding protein
ROS: Reactive oxygen species
RP: Ribosomal protein
RRM: RNA-recognition motif
SAHY: SALT HYPERSENSITIVE MUTANT
SnRK: SNF1-related protein kinase
SOS: Salt overly sensitive
TAIR: The Arabidopsis Information Resource
UTR: Untranslated region
