# Supplementary material for: Salt hypersensitive mutant 9, a nucleolar APUM23 protein, is essential for salt sensitivity in association with the ABA signaling pathway in Arabidopsis
Source: BMC Plant Biol. 2018 Mar 1;18:40. doi: 10.1186/s12870-018-1255-z (PMC5831739; doi:10.1186/s12870-018-1255-z)
Supplement: Supplementary file 1 — Figure S1. SAHY9/APUM23 gene structure showing the T-DNA insertion sites. a: Exon-intron structure of SAHY9/APUM23 and T-DNA insertion sites in the mutant lines. b: Phenotypic comparison between the wild-type and mutant lines. Plants were grown in soil for 35 days. c: RT-PCR analysis of the APUM23 transcript in the wild-type and mutant plants. (PPTX 531 kb) [file 12870_2018_1255_MOESM1_ESM.pptx]

## Slide 1
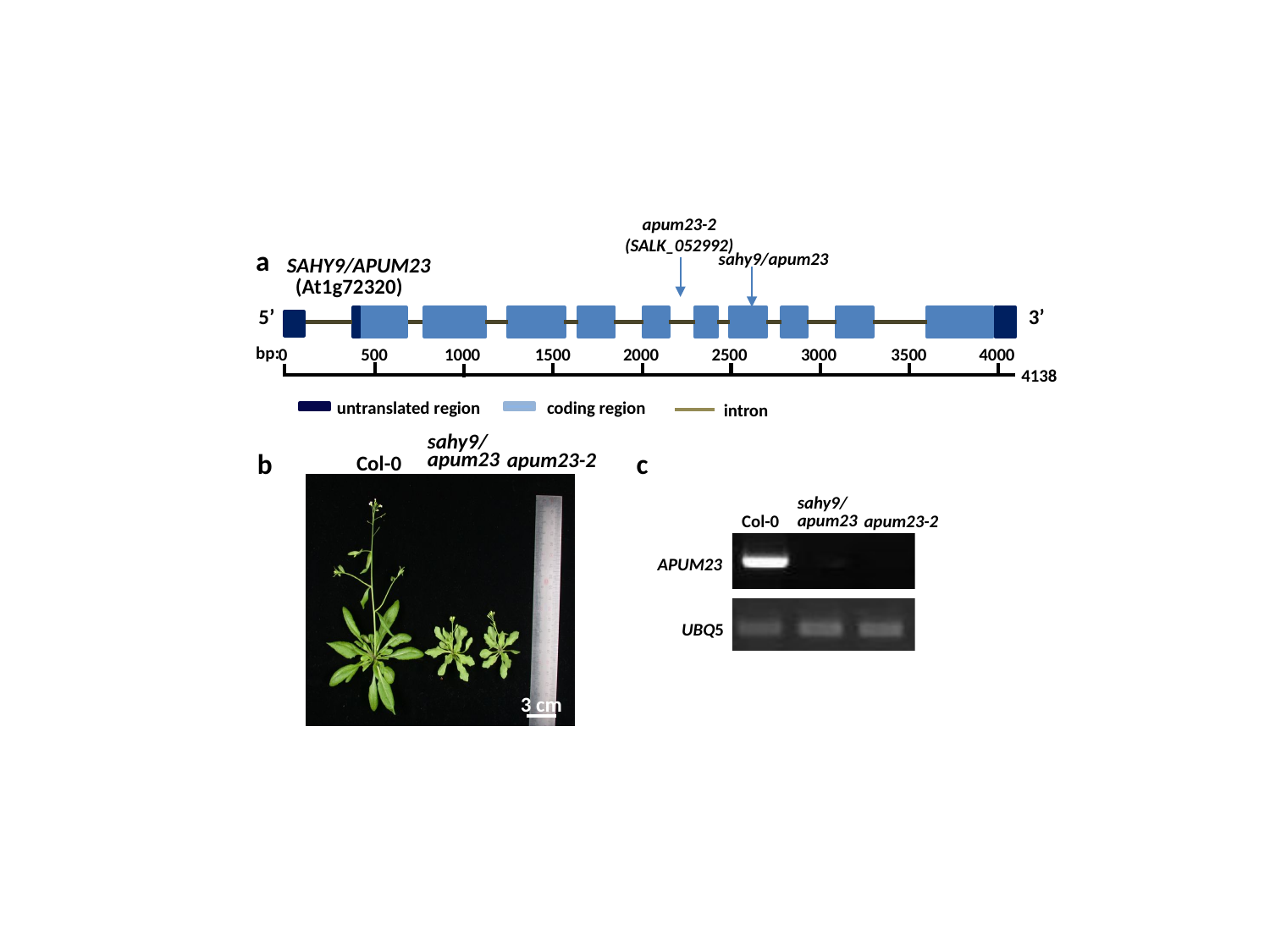

apum23-2
(SALK_052992)
a
sahy9/apum23
SAHY9/APUM23
(At1g72320)
5’
3’
bp:
0
500
1000
1500
2000
2500
3000
3500
4000
4138
untranslated region
coding region
intron
sahy9/ apum23
c
b
 apum23-2
 Col-0
3 cm
sahy9/ apum23
Col-0
apum23-2
APUM23
UBQ5
