# Supplementary figures and images for: Salt hypersensitive mutant 9, a nucleolar APUM23 protein, is essential for salt sensitivity in association with the ABA signaling pathway in Arabidopsis
Source: BMC Plant Biol. 2018 Mar 1;18:40. doi: 10.1186/s12870-018-1255-z (PMC5831739; doi:10.1186/s12870-018-1255-z)

## Slide 1
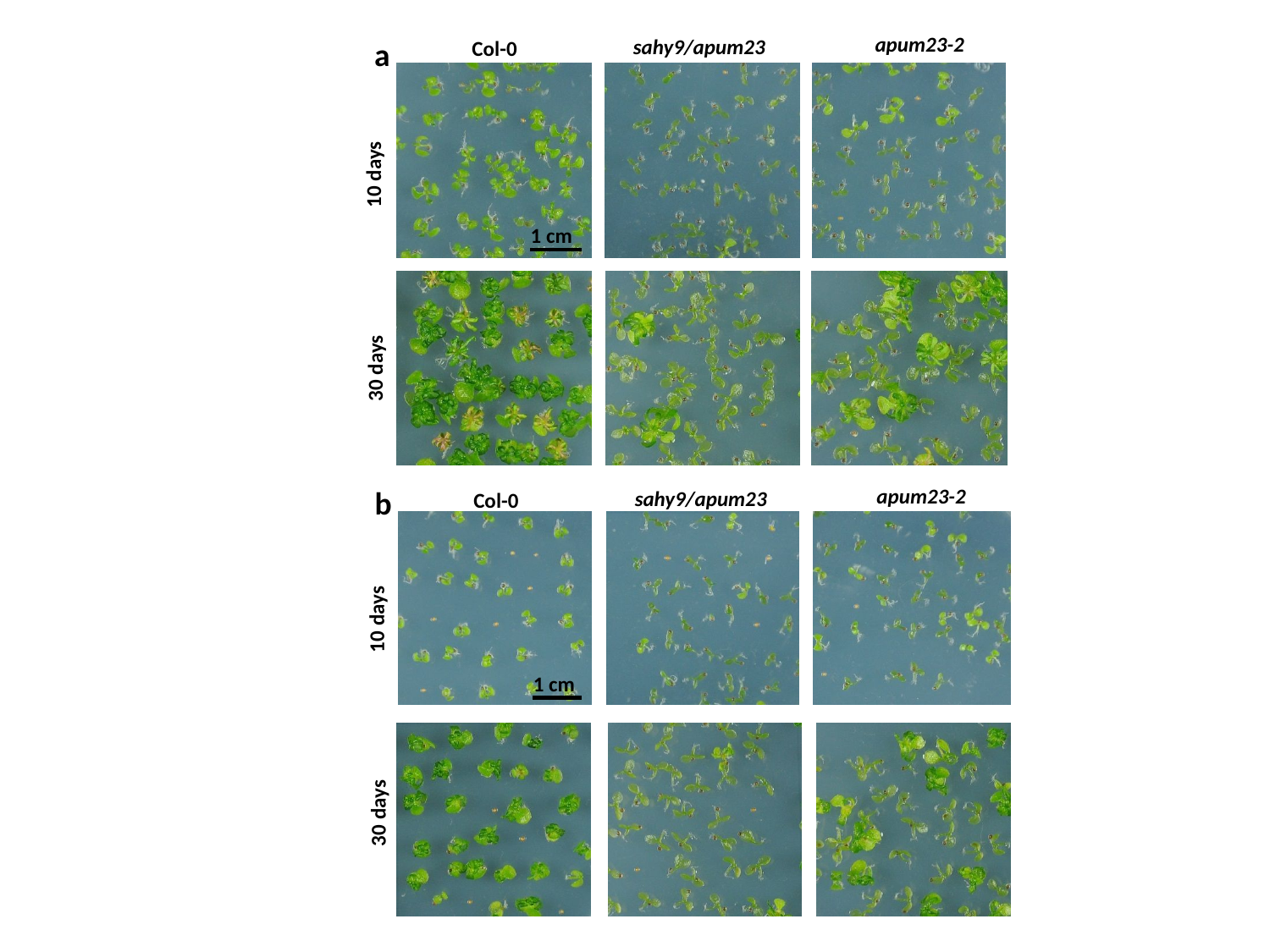

apum23-2
sahy9/apum23
Col-0
10 days
1 cm
30 days
a
apum23-2
b
sahy9/apum23
Col-0
10 days
1 cm
30 days

Supplement: Supplementary file 2 — Figure S2. Effect of osmotic stress on sahy9/apum23 mutant plants. a-b: Seedlings were grown on basal medium supplemented with 4% (a) or 6% (b) mannitol for 10 or 30 days, respectively. (PPTX 482 kb) [file 12870_2018_1255_MOESM2_ESM.pptx]
