# Supplementary material for: Salt hypersensitive mutant 9, a nucleolar APUM23 protein, is essential for salt sensitivity in association with the ABA signaling pathway in Arabidopsis
Source: BMC Plant Biol. 2018 Mar 1;18:40. doi: 10.1186/s12870-018-1255-z (PMC5831739; doi:10.1186/s12870-018-1255-z)
Supplement: Supplementary file 3 — Figure S3. qRT-PCR results of stress-responsive genes and proline contents. a: qRT-PCR of the P5CS1 gene and proline contents. b: qRT-PCR of stress-responsive genes. Plants were grown on basal medium for 10 days, followed by treatment with 150 mM NaCl for 1 day. *, P < 0.05; **, P < 0.01, Student’s t-test. (PPTX 2453 kb) [file 12870_2018_1255_MOESM3_ESM.pptx]

## Slide 1
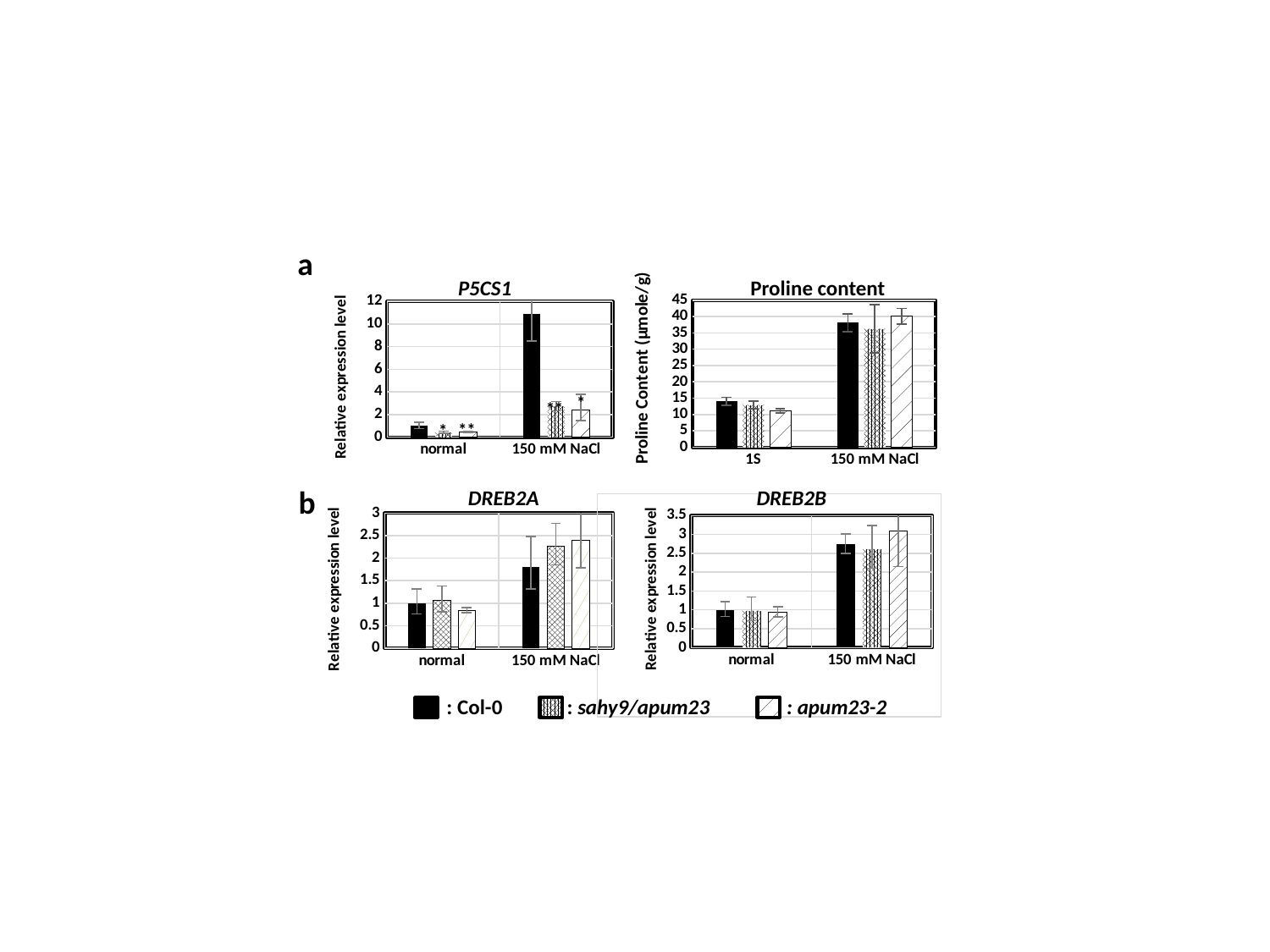

### Chart
| Category | | | |
|---|---|---|---|
| 1S | 14.055752104859828 | 12.943368909848973 | 11.140106360337986 |
| 150 mM NaCl | 38.10052812880904 | 36.25099040989931 | 40.071836383328446 |Proline content
a
### Chart
| Category | | | |
|---|---|---|---|
| normal | 1.0 | 0.4276986820458303 | 0.44927374703563394 |
| 150 mM NaCl | 10.890512931995813 | 2.7182283671437824 | 2.3756681646375206 |P5CS1
*
**
**
*
b
DREB2A
### Chart
| Category | | | |
|---|---|---|---|
| normal | 1.0 | 1.0539290712396865 | 0.8428415447546996 |
| 150 mM NaCl | 1.8027785907023988 | 2.2657677705915966 | 2.3982798275822264 |DREB2B
### Chart
| Category | | | |
|---|---|---|---|
| normal | 1.0 | 0.9787416503974341 | 0.9430023905658058 |
| 150 mM NaCl | 2.747272466859235 | 2.611719574177838 | 3.089414256171764 |: Col-0
: sahy9/apum23
: apum23-2
