# Supplementary material for: Salt hypersensitive mutant 9, a nucleolar APUM23 protein, is essential for salt sensitivity in association with the ABA signaling pathway in Arabidopsis
Source: BMC Plant Biol. 2018 Mar 1;18:40. doi: 10.1186/s12870-018-1255-z (PMC5831739; doi:10.1186/s12870-018-1255-z)
Supplement: Supplementary file 4 — Figure S4. Validation of the expression of genes involved in ribosome biogenesis in sahy9/apum23 under salt stress conditions. The verified genes are listed in Table 2. (PPTX 12701 kb) [file 12870_2018_1255_MOESM4_ESM.pptx]

## Slide 1
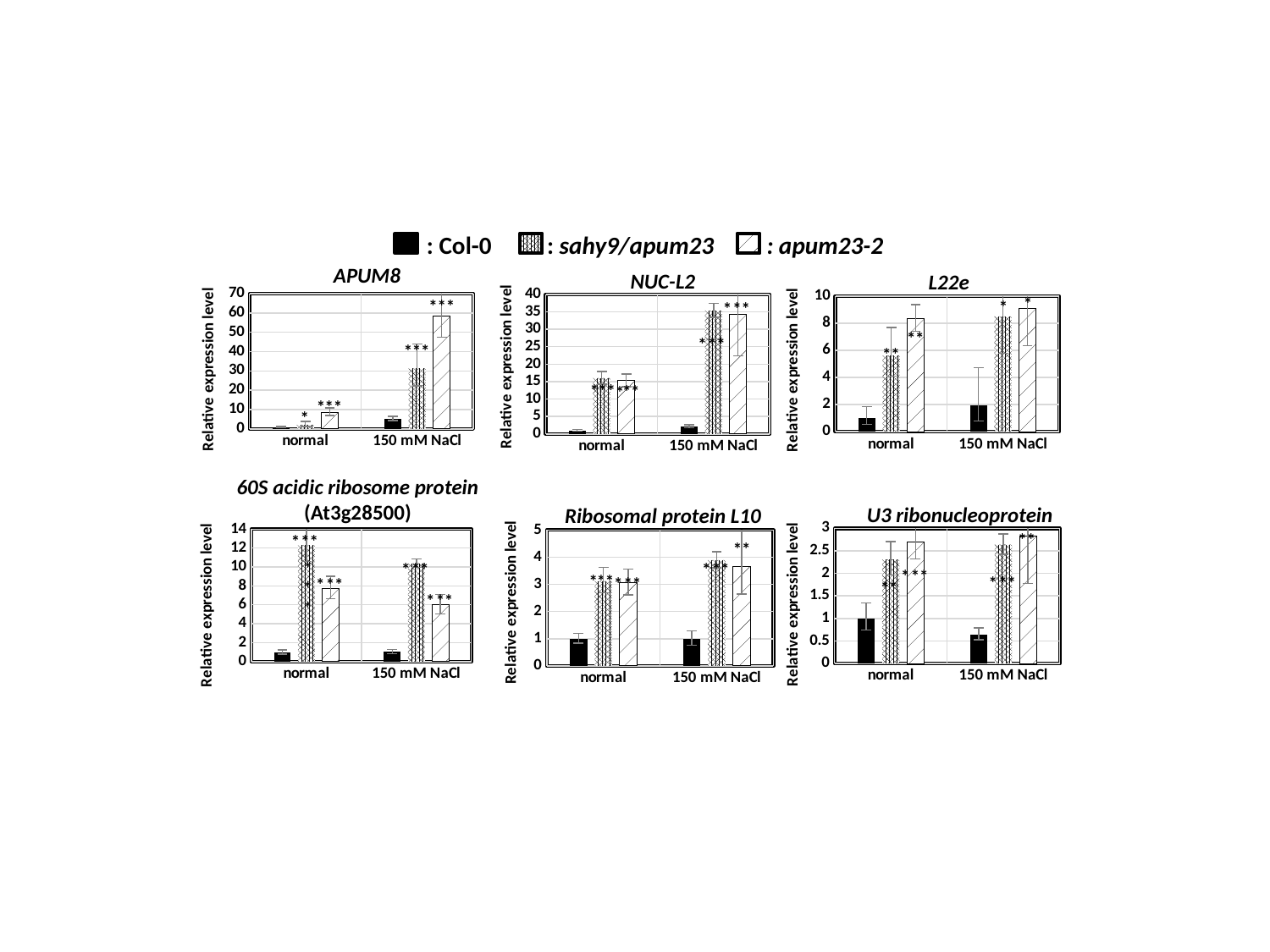

: Col-0
: sahy9/apum23
: apum23-2
APUM8
### Chart
| Category | | | |
|---|---|---|---|
| normal | 1.0 | 2.1145245836043545 | 8.621865160349389 |
| 150 mM NaCl | 5.306154438750507 | 31.261895123906363 | 58.24921356552347 |*
**
*
**
*
**
*
NUC-L2
### Chart
| Category | | | |
|---|---|---|---|
| normal | 1.0 | 16.02219609138146 | 15.252776724610593 |
| 150 mM NaCl | 2.204319478864372 | 35.33436310921907 | 34.24923834965212 |*
**
*
**
*
**
*
**
### Chart
| Category | | | |
|---|---|---|---|
| normal | 1.0 | 5.613886904156304 | 8.330097220278471 |
| 150 mM NaCl | 1.926523788040293 | 8.491385671598346 | 9.075643879487668 |L22e
*
**
**
*
60S acidic ribosome protein
(At3g28500)
### Chart
| Category | | | |
|---|---|---|---|
| normal | 1.0 | 12.280805908898772 | 7.731062315465446 |
| 150 mM NaCl | 1.0343085778843286 | 10.393910718886398 | 5.971113533187839 |*
**
*
**
*
**
*
**
U3 ribonucleoprotein
**
*
**
*
**
**
### Chart
| Category | | | |
|---|---|---|---|
| normal | 1.0 | 2.3075042952320564 | 2.6926001385535616 |
| 150 mM NaCl | 0.6476711259459725 | 2.640235587600648 | 2.827120415825351 |Ribosomal protein L10
### Chart
| Category | | | |
|---|---|---|---|
| normal | 1.0 | 3.1231459704295736 | 3.058165887776932 |
| 150 mM NaCl | 0.9917167307382954 | 3.892418053196134 | 3.6570153104290832 |**
*
**
*
*
**
**
