# Supplementary material for: Salt hypersensitive mutant 9, a nucleolar APUM23 protein, is essential for salt sensitivity in association with the ABA signaling pathway in Arabidopsis
Source: BMC Plant Biol. 2018 Mar 1;18:40. doi: 10.1186/s12870-018-1255-z (PMC5831739; doi:10.1186/s12870-018-1255-z)
Supplement: Supplementary file 5 — Table S1. Overlap of differential gene expression at both the transcript and protein levels in the sahy9/apum23 mutants under salt stress conditions. (DOCX 26 kb) [file 12870_2018_1255_MOESM5_ESM.docx]

**Additional file 5: Table S1.** Overlap of differential gene expression at both the transcript and protein levels in the *sahy9/apum23*^a^ mutants under salt stress conditions.

| Locus | Gene name | Fold change^b^ (Microarray) | Fold change (iTRAQ ) | Biological/molecular function |
| --- | --- | --- | --- | --- |
| AT1G30170 | Hypothetical protein | 257.19 | 2.74 |  |
| AT2G18720 | Translation elongation factor EF1A/  initiation factor IF2gamma family protein | 39.04 | 3.76 | translation |
| AT5G60250 | Zinc finger (C3HC4-type RING finger)  family protein | 27.04 | 2.06 | metal ion binding, nucleic acid binding |
| AT3G18610 | NUC-L2, NUCLEOLIN LIKE 2 | 19.76 | 3.59 | rRNA processing |
| AT3G24780 | Uncharacterised conserved protein | 15.69 | 2.30 |  |
| AT5G13210 | Uncharacterised conserved protein | 14.62 | 2.67 |  |
| AT1G21240 | WAK3, WALL ASSOCIATED KINASE 3 | 12.61 | 0.48 | protein phosphorylation |
| AT3G28500 | 60S acidic ribosomal protein family | 11.41 | 5.37 | structural constituent of ribosome |
| AT2G17850 | Rhodanese/Cell cycle control phosphatase superfamily protein | 10.82 | 2.18 | aging |
| AT5G40040 | 60S acidic ribosomal protein family | 8.98 | 3.13 | structural constituent of ribosome |
| AT1G27020 | Unknown protein | 5.2 | 1.67 |  |
| AT4G12500 | Bifunctional inhibitor/lipid-transfer protein/seed storage 2S albumin  superfamily protein | 5 | 2.11 | lipid transport |
| AT4G12480 | EARLI1, EARLY ARABIDOPSIS ALUMINUM INDUCED 1 | 4.99 | 1.98 | response to salt stress |
| AT4G12470 | AZI1, AZELAIC ACID INDUCED 1 | 4.75 | 1.60 | cold acclimation |
| AT3G57240 | BETA-1,3-GLUCANASE 3, BG3 | 4.71 | 1.73 | carbohydrate metabolic process |
| AT3G27620 | AOX1C, ALTERNATIVE OXIDASE 1C | 4.45 | 0.54 | oxidation-reduction process |
| AT1G33790 | Jacalin lectin family protein | 4.14 | 1.97 | carbohydrate binding |
| AT4G22470 | Protease inhibitor/seed storage/lipid transfer protein (LTP) family protein | 3.96 | 2.0 | lipid transport |
| AT2G18660 | PNP-A, PLANT NATRIURETIC PEPTIDE A | 3.93 | 0.40 | systemic acquired resistance |
| AT4G12490 | AZI3 | 3.85 | 2.11 | defense response to fungus, lipid transport |
| AT4G02390 | PP, POLY(ADP-RIBOSE) POLYMERASE 2 | 3.57 | 1.78 | DNA ligation involved in DNA repair |
| AT1G28660 | GDSL-motif esterase/acyltransferase/lipase | 3.09 | 1.54 | lipid catabolic process |
| AT3G44990 | XTR8, XYLOGLUCAN ENDO-TRANSGLYCOSYLASE-  RELATED 8 | 3.079 | 1.86 | cell wall biogenesis |
| AT1G43800 | FTM1, FLORAL TRANSITION AT THE MERISTEM1 | 3.06 | 1.66 | fatty acid biosynthetic process |
| AT3G21670 | NPF6.4, NRT1/ PTR FAMILY 6.4 | -3.04 | 0.66 | nitrate assimilation, oligopeptide transport |
| AT3G09640 | APX2, ASCORBATE PEROXIDASE 2 | -3.13 | 0.62 | oxidation-reduction process |
| AT1G65450 | GLC, GLAUCE | -3.23 | 0.61 | double fertilization forming a zygote and endosperm |
| AT4G17030 | EXLB1, EXPANSIN-LIKE B1 | -3.26 | 0.59 | plant-type cell wall loosening |
| AT1G70850 | MLP34, MLP-LIKE PROTEIN 34 | -3.27 | 0.61 | defense response |
| AT3G16410 | NSP4, NITRILE SPECIFIER PROTEIN 4 | -3.28 | 0.65 | glucosinolate catabolic process, nitrile biosynthetic process |
| AT1G52560 | HSP20-like chaperones superfamily protein | -3.37 | 0.56 | response to heat |
| AT5G57050 | ABI2 | -3.37 | 0.54 | abscisic acid-activated signaling pathway |
| AT1G72340 | NagB/RpiA/CoA transferase-like  superfamily protein | -3.53 | 0.42 | translational initiation (Chloroplast) |
| AT2G39050 | EULS3, EUONYMUS LECTIN S3 | -3.68 | 0.59 | defense response to bacterium, stomatal closure |
| AT2G22240 | MIPS2, MYO-INOSITOL-1-PHOSPHATE SYNTHASE 2 | -3.71 | 0.53 | defense response |
| AT3G02480 | ABR, ABA-RESPONSE PROTEIN | -3.71 | 0.61 | a LEA protein, response to water deprivation |
| AT5G59720 | HSP18.2 | -3.8 | 0.65 | response to heat |
| AT2G36780 | UDP-Glycosyltransferase superfamily  protein | -3.9 | 0.48 | metabolic process |
| AT3G25830 | TPS-CIN, TERPENE SYNTHASE-LIKE SEQUENCE-1,8-CINEOLE | -4.04 | 0.53 | defense response |
| AT5G28510 | BGLU24 | -4.13 | 0.47 | carbohydrate metabolic process |
| AT3G11410 | PP2CA | -4.14 | 0.50 | negative regulation of abscisic acid-activated signaling pathway |
| AT1G69260 | AFP1, ABI FIVE BINDING PROTEIN | -4.27 | 0.63 | abscisic acid-activated signaling pathway |
| AT2G41190 | Transmembrane amino acid transporter  family protein | -4.33 | 0.46 | amino acid transport |
| AT3G14440 | NCED3 | -4.37 | 0.41 | abscisic acid biosynthetic process |
| AT3G24520 | HSFC1, HEAT SHOCK TRANSCRIPTION FACTOR C1 | -4.53 | 0.64 | regulation of transcription |
| AT4G25000 | AMY1, ALPHA-AMYLASE-LIKE | -4.55 | 0.33 | response to GA and ABA |
| AT5G06760 | LEA4-5 | -4.57 | 0.54 | response to osmotic stress |
| AT2G01520 | MLP328, MLP-LIKE PROTEIN 328 | -4.82 | 0.46 | defense response |
| AT5G54165 | Unknown protein | -5.03 | 0.38 | proteolysis, response to stress |
| AT5G24770 | VSP2 | -5.03 | 0.37 | defense response; response to JA |
| AT4G14060 | Polyketide cyclase/dehydrase and lipid transport superfamily protein | -5.16 | 0.56 | defense response |
| AT3G17520 | Late embryogenesis abundant protein (LEA) family protein | -5.32 | 0.54 | embryo development ending in seed dormancy |
| AT4G10250 | ATHSP22.0 | -5.35 | 0.63 | response to heat |
| AT4G31830 | Unknown protein | -5.39 | 1.71 |  |
| AT2G47770 | TSPO, TSPO(OUTER MEMBRANE TRYPTOPHAN-RICH SENSORY PROTEIN)-RELATED | -5.48 | 0.54 | response to ABA and salt stress |
| AT5G47450 | TIP2;3 | -5.5 | 1.63 | response to salt stress |
| AT2G42540 | COR15A | -5.91 | 0.60 | response to ABA and salt stress |
| AT5G66780 | Unknown protein | -6.18 | 0.54 |  |
| AT2G34810 | FAD-binding Berberine family protein | -6.68 | 0.63 | response to JA and wounding |
| AT5G52300 | RD29B | -6.83 | 0.51 | response ABA and salt stress |
| AT3G55940 | Phosphoinositide-specific phospholipase C family protein | -7.12 | 0.62 | lipid catabolic process |
| AT4G12580 | Unknown protein | -7.15 | 0.58 |  |
| AT2G33380 | RD20 | -7.22 | 0.45 | response ABA and salt stress |
| AT5G05220 | Unknown protein | -7.78 | 0.38 |  |
| AT1G77520 | O-methyltransferase family protein | -8.26 | 0.56 | lignin biosynthetic process, methylation |
| AT5G24780 | VSP1 | -8.52 | 0.50 | defense response |
| AT5G37990 | CIMT1, S-adenosyl-L-methionine-dependent methyltransferases superfamily protein | -10.09 | 0.52 | methylation |
| AT3G16780 | RPL19B, RIBSOMAL PROTEIN LIKE 19B | -4001.88 | 0.35 | ribosome biogenesis (60S) |

^a^Plants were vertically grown on half-strength MS medium for 10 days, then transferred to the fresh media supplemented with or without 150 mM NaCl for one day. ^b^Fold change in *sahy9/apum23* are normalized with the wild type. The microarray raw data are available in GEO database with an accession no. GSE99664.
