# Supplementary material for: Salt hypersensitive mutant 9, a nucleolar APUM23 protein, is essential for salt sensitivity in association with the ABA signaling pathway in Arabidopsis
Source: BMC Plant Biol. 2018 Mar 1;18:40. doi: 10.1186/s12870-018-1255-z (PMC5831739; doi:10.1186/s12870-018-1255-z)
Supplement: Supplementary file 6 — Figure S5. Expression of genes regulated by ABA and salt stress. Plants were grown on basal medium supplemented with 150 mM NaCl or 150 mM NaCl + 50 nM ABA for 24 days. qRT-PCR was performed for the detection of relative gene expression levels. (PPTX 29522 kb) [file 12870_2018_1255_MOESM6_ESM.pptx]

## Slide 1
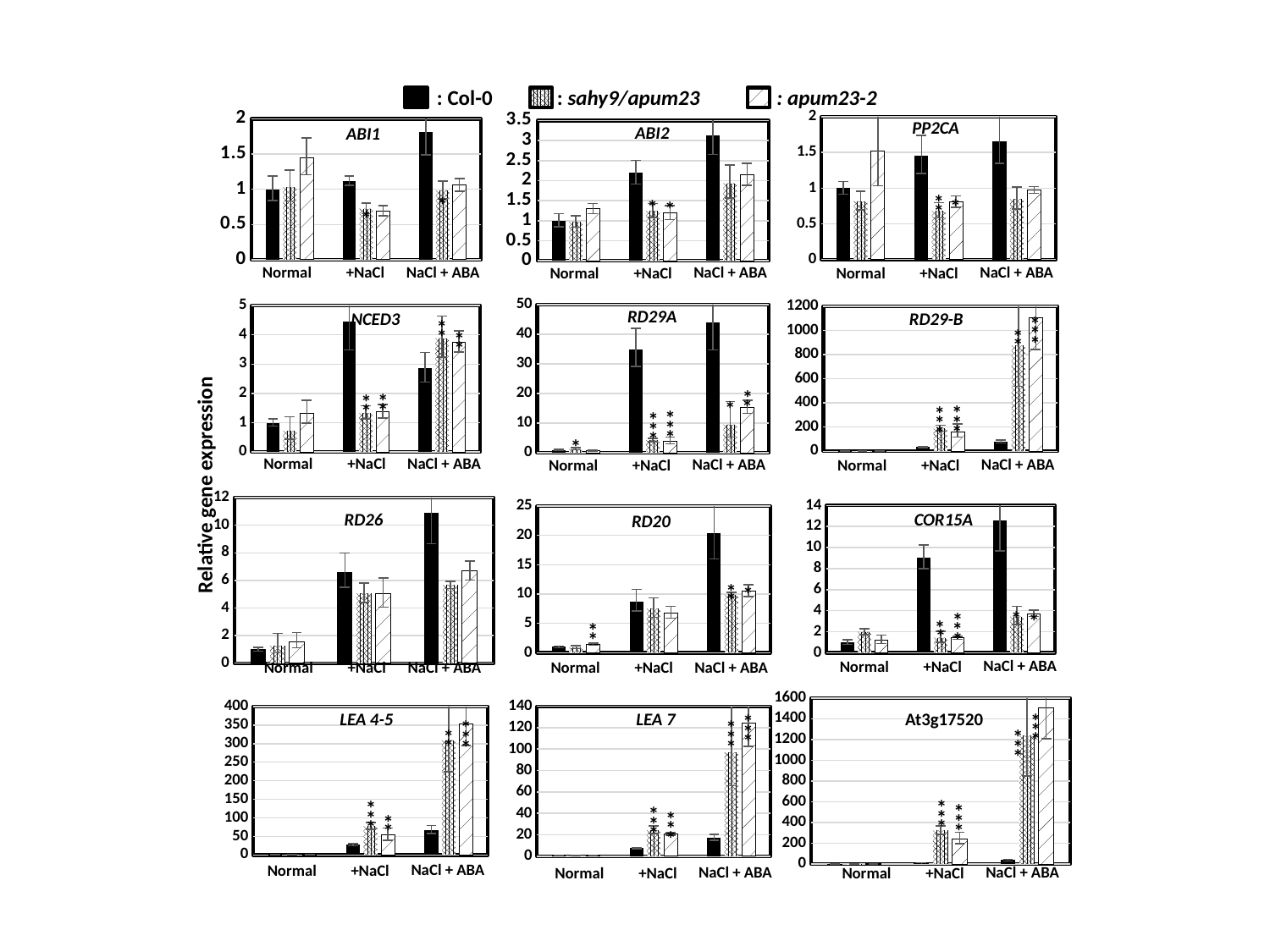

: Col-0
: sahy9/apum23
: apum23-2
### Chart
| Category | Col-0 | sahy9-1 | sahy9-2 |
|---|---|---|---|
| normal | 1.0 | 0.8154490628319042 | 1.5230887397032606 |
| 150 mMNaCl | 1.450281880436632 | 0.6804998112427048 | 0.8068281545749052 |
| 150 mM NaCl+ 50 nM ABA | 1.6605563338747542 | 0.8455722873775181 | 0.974454458092832 |*
*
*
PP2CA
NaCl + ABA
Normal
+NaCl
### Chart
| Category | Col-0 | sahy9-1 | sahy9-2 |
|---|---|---|---|
| normal | 1.0 | 1.0302539536200714 | 1.441928871460227 |
| 150 mMNaCl | 1.1206481089767066 | 0.7248059313622777 | 0.6915154228965676 |
| 150 mM NaCl+ 50 nM ABA | 1.815877230242735 | 0.9819126990460242 | 1.0592183346838753 |ABI1
*
*
NaCl + ABA
Normal
+NaCl
### Chart
| Category | Col-0 | sahy9-1 | sahy9-2 |
|---|---|---|---|
| normal | 1.0 | 0.9661595333904439 | 1.2917557747834172 |
| 150 mMNaCl | 2.1936499593892567 | 1.2417143553198495 | 1.187559666073414 |
| 150 mM NaCl+ 50 nM ABA | 3.13906158503524 | 1.934999687442399 | 2.1415667822238014 |ABI2
*
*
NaCl + ABA
Normal
+NaCl
### Chart:
| Category | Col-0 | sahy9-1 | sahy9-2 |
|---|---|---|---|
| normal | 1.0 | 1.6525187792853673 | 2.6585998783917244 |
| 150 mMNaCl | 33.79333119177129 | 188.8373065066021 | 162.65447072664693 |
| 150 mM NaCl+ 50 nM ABA | 80.56045910431054 | 874.2083411950276 | 1104.8744044943487 |*
*
*
*
*
*
*
*
*
*
*
RD29-B
NaCl + ABA
Normal
+NaCl
### Chart
| Category | Col-0 | sahy9-1 | sahy9-2 |
|---|---|---|---|
| normal | 1.0 | 1.2884768947149678 | 0.7865806856284958 |
| 150 mMNaCl | 34.94465746386109 | 4.277199994326967 | 3.9494908142134033 |
| 150 mM NaCl+ 50 nM ABA | 44.037683210047035 | 9.518054178906748 | 15.394395724427758 |RD29A
*
*
*
*
*
*
*
*
*
*
NaCl + ABA
Normal
+NaCl
### Chart
| Category | Col-0 | sahy9-1 | sahy9-2 |
|---|---|---|---|
| normal | 1.0 | 0.7261468961291594 | 1.3243948752388326 |
| 150 mMNaCl | 4.440329282077371 | 1.3308364940821773 | 1.3679353039024926 |
| 150 mM NaCl+ 50 nM ABA | 2.8500752282975736 | 3.8807441800863893 | 3.7537519866845863 |NCED3
*
*
*
*
*
*
*
*
NaCl + ABA
Normal
+NaCl
Relative gene expression
### Chart
| Category | Col-0 | sahy9-1 | sahy9-2 |
|---|---|---|---|
| normal | 1.0 | 1.2567229763124066 | 1.560130452026746 |
| 150 mMNaCl | 6.625365290676355 | 5.060687301392006 | 5.01181559601555 |
| 150 mM NaCl+ 50 nM ABA | 10.880452610455999 | 5.663393080031554 | 6.680703355426947 |RD26
### Chart
| Category | Col-0 | sahy9-1 | sahy9-2 |
|---|---|---|---|
| normal | 1.0 | 1.98549675088063 | 1.2229228002162063 |
| 150 mMNaCl | 9.050515702767251 | 1.438268805115604 | 1.4237212081453094 |
| 150 mM NaCl+ 50 nM ABA | 12.599838469657316 | 3.44379675335563 | 3.7299768246782543 |COR15A
*
*
*
*
*
*
*
NaCl + ABA
Normal
+NaCl
### Chart
| Category | Col-0 | sahy9-1 | sahy9-2 |
|---|---|---|---|
| normal | 1.0 | 0.9272305464354592 | 1.426684833444155 |
| 150 mMNaCl | 8.750305176120587 | 7.5179188011340505 | 6.808482747875605 |
| 150 mM NaCl+ 50 nM ABA | 20.37883960522399 | 9.851431207748814 | 10.551186476049253 |RD20
*
*
*
*
*
NaCl + ABA
Normal
+NaCl
NaCl + ABA
Normal
+NaCl
### Chart
| Category | Col-0 | sahy9-1 | sahy9-2 |
|---|---|---|---|
| normal | 1.0 | 0.5475262353967848 | 0.9354073478731165 |
| 150 mMNaCl | 7.4625395203474225 | 24.459675064355363 | 20.81906443550489 |
| 150 mM NaCl+ 50 nM ABA | 17.464256666851487 | 96.9610443864376 | 124.21250963117316 |
### Chart
| Category | Col-0 | sahy9-1 | sahy9-2 |
|---|---|---|---|
| normal | 1.0 | 0.41571498753322633 | 0.7822310271120595 |
| 150 mMNaCl | 27.499497157328143 | 77.56496951065205 | 53.717986831469894 |
| 150 mM NaCl+ 50 nM ABA | 67.85265361830307 | 309.0793231144249 | 352.7901979007491 |LEA 4-5
*
*
*
*
*
*
*
*
*
*
### Chart
| Category | Col-0 | sahy9-1 | sahy9-2 |
|---|---|---|---|
| normal | 1.0 | 0.4751099524648656 | 1.421748878892979 |
| 150 mMNaCl | 11.524774894654055 | 321.8697829978194 | 244.1008203439046 |
| 150 mM NaCl+ 50 nM ABA | 36.74136532066834 | 1235.7461245386528 | 1504.776317468788 |*
*
*
LEA 7
At3g17520
*
*
*
*
*
*
*
*
*
*
*
*
*
*
*
*
*
*
*
*
*
NaCl + ABA
Normal
+NaCl
NaCl + ABA
Normal
+NaCl
NaCl + ABA
Normal
+NaCl
