# Supplementary material for: Salt hypersensitive mutant 9, a nucleolar APUM23 protein, is essential for salt sensitivity in association with the ABA signaling pathway in Arabidopsis
Source: BMC Plant Biol. 2018 Mar 1;18:40. doi: 10.1186/s12870-018-1255-z (PMC5831739; doi:10.1186/s12870-018-1255-z)
Supplement: Supplementary file 7 — Figure S6. ABA contents. Seedlings were grown on basal medium supplemented with NaCl or NaCl + ABA for 24 days. The values indicate the means ± SD of four independent experiments. ***, P < 0.001, Student’s t-test. (PPTX 88 kb) [file 12870_2018_1255_MOESM7_ESM.pptx]

## Slide 1
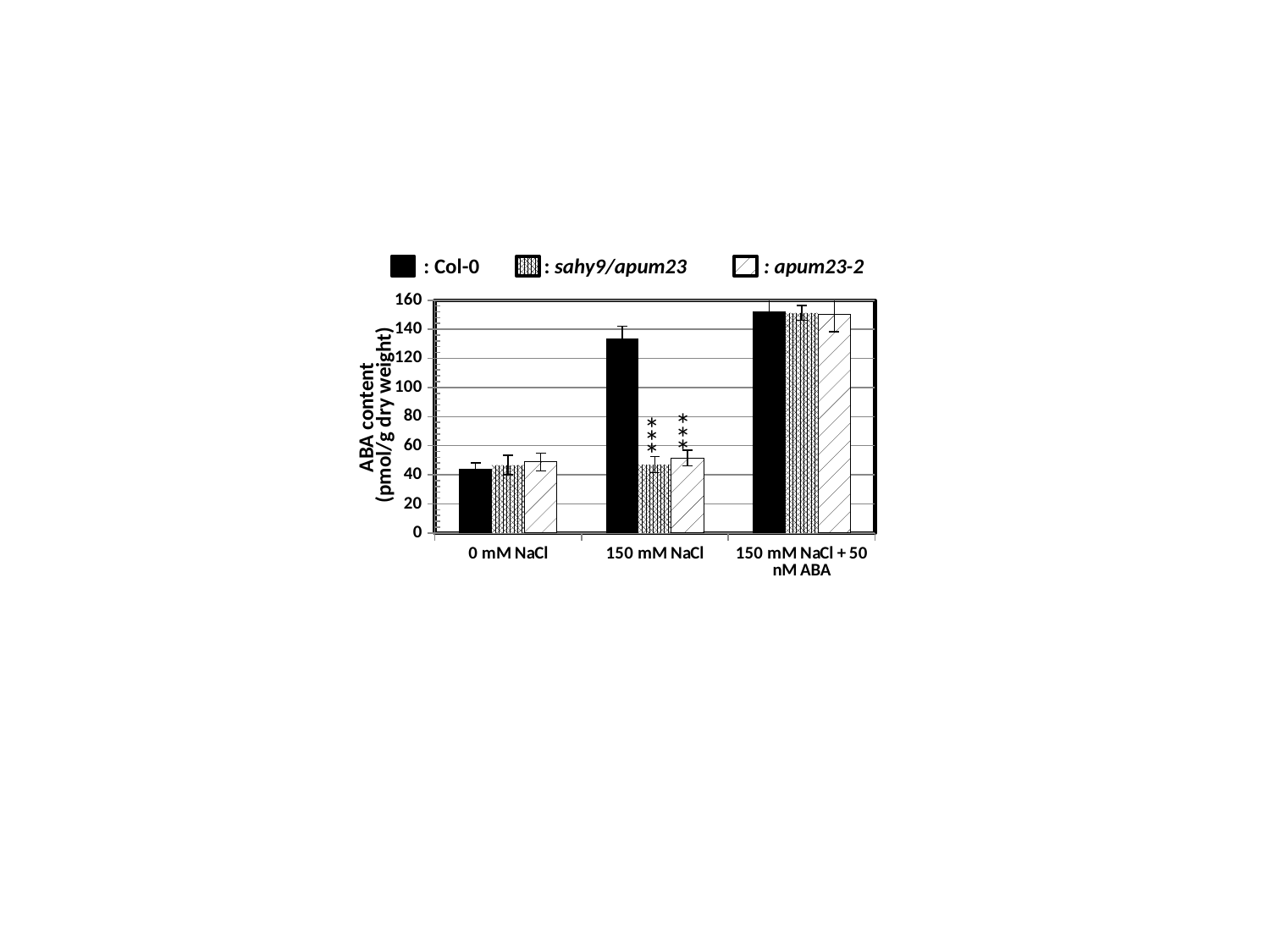

: Col-0
: sahy9/apum23
: apum23-2
### Chart
| Category | Col-0 | sahy9/apum23 | apum23-2 |
|---|---|---|---|
| 0 mM NaCl | 43.93207782114034 | 46.654968839270666 | 48.731754463238374 |
| 150 mM NaCl | 133.63853422277603 | 46.92705188577746 | 51.47769367054889 |
| 150 mM NaCl + 50 nM ABA | 152.29302857293814 | 151.24327573514336 | 150.29964476727838 |ABA content
 (pmol/g dry weight)
*
*
*
*
*
*
