# Supplementary material for: Salt hypersensitive mutant 9, a nucleolar APUM23 protein, is essential for salt sensitivity in association with the ABA signaling pathway in Arabidopsis
Source: BMC Plant Biol. 2018 Mar 1;18:40. doi: 10.1186/s12870-018-1255-z (PMC5831739; doi:10.1186/s12870-018-1255-z)
Supplement: Supplementary file 8 — Table S2. Primer sequences used in this study. (DOCX 16 kb) [file 12870_2018_1255_MOESM8_ESM.docx]

| **Table S2.** Primer sequences used in this study | | | |
| --- | --- | --- | --- |
| AGI number | Description | Sequence (5'→3') | |
| At3g25800 | PP2A | F | TAAGCTTGGTGCTCTTTGCATG |
|  |  | R | GCATCGCGGATTGAGTGAA |
| At3g14440 | NCED3 | F | TCCAGATTGCTTCTGCTTCCAT |
|  |  | R | GGACCCTATCACGACGACTTCA |
| At5g57050 | ABI2 | F | GCGTTGTCGGTTGATCACAA |
|  |  | R | TTCTATCCTCGCCGCTTCA |
| At4g26080 | ABI1 | F | CTGCACTTCCATTATCCGTTGA |
|  |  | R | TCAATCCTCGCAGCTTCATCT |
| At3g11410 | PP2CA | F | GCGGCGTTGCTCTTAACAAA |
|  |  | R | GCTCACGTTATCGGAGCTCTGT |
| At2g42540 | COR15A | F | CAAACAAGGCGGCAGAGTT |
|  |  | R | CATCCTTAGCCTCTCCTGCTTT |
| At5g05410 | DREB2A | F | AACAGTGTTGCCAACGGTTCA |
|  |  | R | TGAGGCTTTGTAGCGGATCAA |
| At3g11020 | DREB2B | F | AGCCAAGACCAAAACCACGTAA |
|  |  | R | CGAGATGAAGCGGATGCAA |
| At2g33380 | RD20 | F | TGTGAGAGGTTGCTTTGATGGA |
|  |  | R | CCCTCTCTTTGGCAATTTGT |
| At4g27410 | RD26 | F | CTTGATTCGTTCCCGGAGATAA |
|  |  | R | GCGAATTCATCCGAGGAAGA |
| AT5G52310 | RD29A | F | TGCACCAGGCGTAACAGGTAA |
|  |  | R | ACACCTTTGTCCCTGGTGGAAT |
| At5g52300 | RD29B | F | CCTGTGTCTCTGCTTTCAGCAA |
|  |  | R | CACCAGGAGCAAACGTCCTAGT |
| At4g04490 | CPK36 | F | GCCTCCTCGATCTCCTCAAACT |
|  |  | R | CGCTCCATGGTTGAAACATTCT |
| At4g12480 | EARLI1 | F | TCGGTGTATGTGCGAACGTT |
|  |  | R | TGATGGCTGACCCAATTGAA |
| At2g18190 | P-loop containing nucleoside triphosphate hydrolases superfamily protein | F | ATGTGCGTGCGAGCAAAGAT |
|  |  | R | CGAATGTCGATGGATGCTCAA |
| At5g06760 | LEA4-5 | F | GGCATGGACAAAACCAAAGCT |
|  |  | R | CGCGTTTCTCTCTTCTGCATCT |
| At1g52690 | LEA7 | F | GGCACACGATGCAGCTGAATA |
|  |  | R | CCCAAGATCCCACTGGTCTTCT |
| At3g17520 |  | F | GAAGTTGGCGGAGACAGTTGA |
|  |  | R | TCGTCATATCGCTCGCCTTCT |
| At2g39800 | P5CS1 | F | GGAAAGATCTCGAGCCTTGCA |
|  |  | R | GCCGATTGGATCTTCCATATCA |
| At1g22240 | APUM8 | F | ATCCAAGCTGCTCTTGCTGTT |
|  |  | R | AACGAGTGTGGCATGAAGTGA |
| At3g18610 | NUC-L2 | F | GAAGCTCAGAAGGCATTGGAAA |
|  |  | R | CATCGCGGCCTAGTAACAATTT |
| At1g02830 | Ribosomal L22e protein family | F | GGAGATAGCCACGCTTGAGAA |
|  |  | R | CCTCTGACCTTGATTCGTTCCT |
| At2g40010 | Ribosomal protein L10 family protein | F | CAATTGATGTGGTCGTGCAA |
|  |  | R | CTGGAAGAAGGAGGTCTGTGAA |
| At3g28500 | 60S acidic ribosomal protein family | F | GCCGGTTGCTGAATCTAAGAA |
|  |  | R | TGCCTGCATCGTCAGAAGA |
| At5g08600 | U3 ribonucleoprotein (Utp) family protein | F | TGAAGTCCCCATTTCTCGTCTT |
|  |  | R | TCACAGCACGAGCCATGAA |
